# Notch and Hippo signaling converge on Strawberry Notch 1 (Sbno1) to synergistically activate *Cdx2* during specification of the trophectoderm

**DOI:** 10.1038/srep46135

**Published:** 2017-04-12

**Authors:** Yusuke Watanabe, Kota Y. Miyasaka, Atsushi Kubo, Yasuyuki S. Kida, Osamu Nakagawa, Yoshikazu Hirate, Hiroshi Sasaki, Toshihiko Ogura

**Affiliations:** 1 Department of Developmental Neurobiology, Institute of Development, Aging and Cancer, Tohoku University, 4-1 Seiryo, Aoba, Sendai, Miyagi 980-8575, Japan; 2 Department of Molecular Physiology, National Cerebral and Cardiovascular Center Research Institute, 5-7-1 Fujishiro-dai, Suita, Osaka, 565-8565, Japan; 3 Biotechnology Research Institute for Drug Discovery, National Institute of Advanced Industrial Science and Technology (AIST), 1-1-1 Umezono, Tsukuba, Ibaraki 305-8568, Japan; 4 Department of Cell Fate Control, Institute of Molecular Embryology and Genetics, Kumamoto University, 2-2-1 Honjo, Chuo-ku, Kumamoto 860-0811, Japan; 5 Laboratory for Embryogenesis, Graduate School of Frontier Biosciences, Osaka University, 1-3 Yamadaoka, Suita, Osaka 585-0871, Japan

## Abstract

The first binary cell fate decision occurs at the morula stage and gives rise to two distinct types of cells that constitute the trophectoderm (TE) and inner cell mass (ICM). The cell fate determinant, Cdx2, is induced in TE cells and plays an essential role in their differentiation and maintenance. Notch and Hippo signaling cascades are assumed to converge onto regulatory elements of *Cdx2,* however, the underlying molecular mechanisms are largely unknown. Here, we show involvement of Strawberry Notch1 (Sbno1), a novel chromatin factor of the helicase superfamily 2, during preimplantation development. *Sbno1* knockout embryos die at the preimplantation stage without forming a blastocoel, and Cdx2 is not turned on even though both Yap and Tead4 reside normally in nuclei. Accordingly, Sbno1 acts on the trophectoderm-enhancer (TEE) of *Cdx2*, ensuring its robust and synergistic activation by the Yap/Tead4 and NICD/Rbpj complexes. Interestingly, this synergism is enhanced when cells are mechanically stretched, which might reflect that TE cells are continuously stretched by the expanding ICM and blastocoel cavity. In addition, the histone chaperone, FACT (FAcilitates Chromatin Transcription) physically interacts with Sbno1. Our data provide new evidence on TE specification, highlighting unexpected but essential functions of the highly conserved chromatin factor, Sbno1.

The correct formation of tissues and organs during the embryonic period is governed by the orchestrated actions of multiple signaling pathways, including Fgf, Wnt, Bmp, Notch and Hippo pathways. In concert with these cascades, transcription factors regulate gene expression in a spatiotemporally controlled manner to achieve correct growth and differentiation of cells. The first sign of the binary differentiation of cell lineages in the preimplantation embryo is specification of the inner cell mass (ICM) and trophectoderm (TE). The ICM is initially maintained in a pluripotent state by Oct3/4 (pou5f1), Nanog and Sox2, and subsequently develops into the epiblast and visceral endoderm, whereas TE cells differentiate into the placenta and ectoplacental cone under the regulatory actions of Cdx2, Eomesodermin and Gata3^1^,^2^. Among various signaling cascades, Hippo signaling has been reported to be responsible for TE cell fate determination. Suppression of Hippo signaling in the outer cell layer leads to nuclear localization of Yap, resulting in direct association of Yap and Tead4 in the nucleus and subsequent transcriptional activation of their targets including *Cdx2*, which is necessary for TE differentiation^3^,^4^. Transcription factors Tfap2c and Gata3 also directly regulate *Cdx2*, either upstream or downstream of Hippo signaling^5^,^6^, and cooperation between Yap/Tead4 and Notch1/Rbpj transcriptional activities on *Cdx2* in the TE was also reported^7^; hence the specification of the TE is controlled by a joint action of different signaling cascades and transcription factors.

In addition to the cell-type specific actions of transcription factors, ATP-dependent helicase-related factors involved in chromatin remodeling have recently been shown to be essential during embryonic development^8^. For example, the helicases or helicase-related enzymes unwind and/or twist DNA/RNA to alter chromatin structures, which is a prerequisite for subsequent events, such as gene transcription or DNA replication and repair. These helicase-like proteins can be classified into six groups, namely helicase superfamily 1 to 6 (SF1 to SF6), based on their sequences and conserved motifs^9^,^10^,^11^. Among them, DExx box helicases and Swi/Snf chromatin remodelers are classified as the SF2 superfamily.

Strawberry Notch (Sbno in vertebrates, Sno in Drosophila) is a helicase-related nuclear factor. The N- and C-terminal regions of Sbno/Sno are highly conserved in both vertebrates and invertebrates^12^,^13^, and these regions contain two characteristic motifs, the DExH box and helicase-c domain, respectively. Based upon these structural features, Sbno/Sno is classified as a helicase-like protein^14^,^15^,^16^ that belongs to the SF2 superfamily. Nonetheless, the molecular functions of Sbno/Sno, especially from a viewpoint of transcriptional control, remain obscure.

Genetic and molecular analyses in fly, worm and fish have revealed that Sbno/Sno is relevant to developmental processes that involve Notch. In Drosophila, *sno* mutants are embryonic lethal with severely impaired cuticular and nervous system development. In contrast, heat-inducible *sno* mutants in eclosed flies phenocopy the *notch* or *Su(H*) mutant, which shows disrupted ommatidia, fused segments of legs and notched wing margin. More importantly, these phenotypes can be rescued by additional notch or Su(H)^17^. In the developing wing margin, Notch-induced *sno* regulates expression of wingless, vestigial, cut and E(spl)-m8^12^,^18^. These lines of evidence suggest that sno acts in the Notch cascade, thereby affecting other signaling pathways, such as Wnt and Hippo^18^, and highlighting its crucial action at the intersection of different signaling pathways. During photoreceptor specification in Drosophila, Sno binds to Su(H) and an F-box/WD40 protein Ebi, which recruit the transcriptional co-repressor SMRTER to keep its direct target *Delta* inactive. This transcriptional repression is relieved by epidermal growth factor receptor (EGFR) signaling, and this de-repression is proteasome-dependent and accompanied by cytoplasmic translocation of SMRTER. This EGFR pathway-regulated *Delta* transcription allows transmission of Delta signal to neighboring Notch-expressing cells, a molecular basis for the binary specification of photoreceptor and non-neuronal cone cells^13^. On the other hand, in *C. elegans, let765*/*sno* functions upstream of the lin-3/egf-Ras pathway to regulate vulval development^15^. In zebrafish, Sbno1 also interacts with Su(H), and is involved in neural development^19^,^20^. These studies indicate that Sbno/Sno acts on different signaling pathways and also in distinct tissue-specific contexts, yet its precise molecular actions are largely unknown.

In this study, we analyzed Sbno1 function during mouse development. When *Sbno1* is disrupted in mouse, embryonic development is arrested at the preimplantation stage with a loss of expression of TE-specific genes. We found that Sbno1 is required for transcriptional activities of Yap/Tead4 and Notch/Rbpj. Furthermore, Sbno1 is indispensable for transcriptional activation of the *Cdx2* TE enhancer, which is regulated by a synergistic action of Yap/Tead4 and Notch/Rbpj. Physical interaction between Sbno1, Yap/Tead4, Rbpj and the FACT complex indicates that Sbno1 regulates activity of these transcription factors on target genes. Our results highlight a critical role of this helicase-related factor on specific gene activation during preimplantation development.

## Results

### *Sbno1* functions during mouse preimplantation development

**Figure 1:**
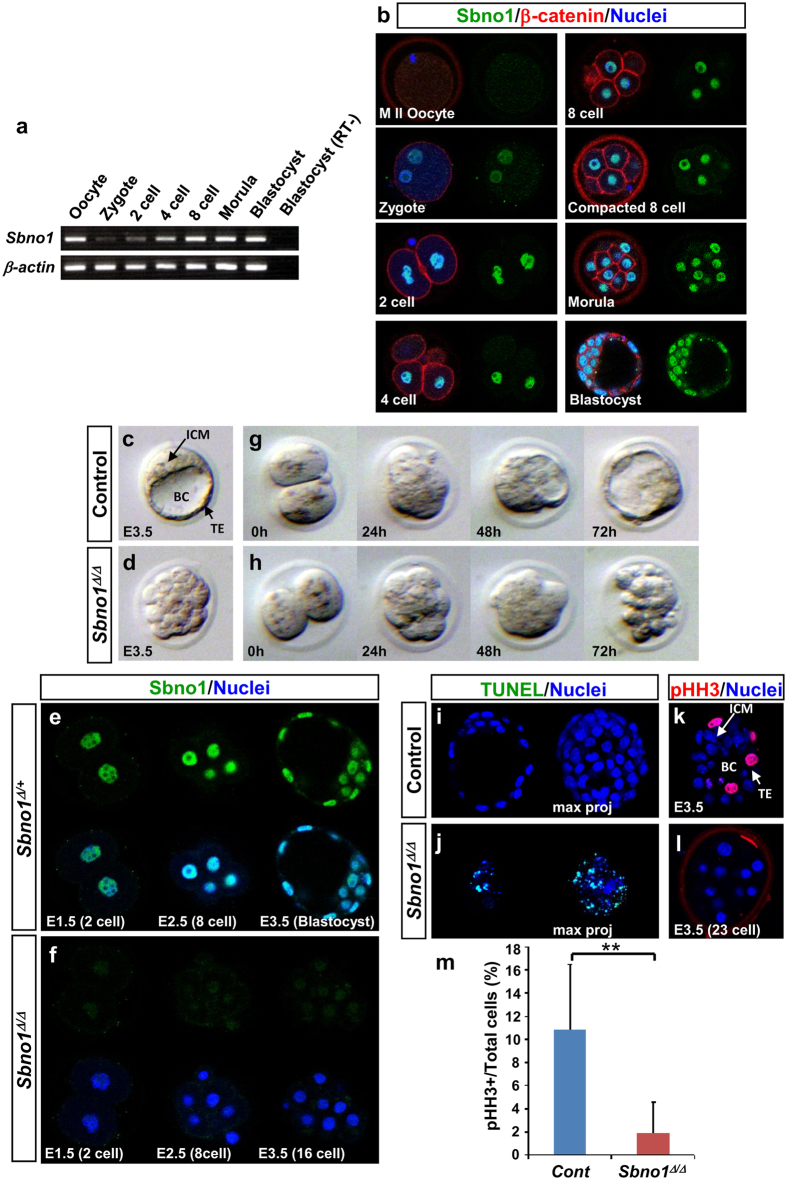
Expression patterns of *Sbno1* during mouse preimplantation development and phenotypes of *Sbno1* knockout embryos (*Sbno1*^Δ/Δ^). (**a**) Expression levels of *Sbno1* transcripts during the preimplantation period were analyzed by semi-quantitative RT-PCR. *β-Actin* was used as an internal control. (**b**) Immunohistochemistry showed that Sbno1 (green) is clearly localized in the nuclei from the two-cell to blastocyst stages. β-Catenin staining (red) demarcates the cell membrane, and DAPI staining (blue) identifies the nuclei. (**c**,**d**) At E3.5, control *Sbno1*^Δ/+^ embryos develop to blastocysts with an inner cell mass (ICM), trophectoderm (TE) and blastocoel (BC), whereas development of *Sbno1*^Δ/Δ^ embryos was halted with a morula-like morphology. (**e**,**f**) In *Sbno1*^Δ/Δ^ embryos, Sbno1 protein (green) was absent from the two-cell stage. (**g**,**h**) When two-cell stage embryos were cultured *in vitro, Sbno1*^Δ/Δ^ embryos developed normally to the compacted morula stage, but failed to form blastocysts and collapsed. (**i**–**m**) TUNEL (green in **i**,**j**) and phospho-histone H3 staining (pHH3, red in **k**,**l**) revealed increased cell death and decreased proliferation in *Sbno1*^Δ/Δ^ embryos, respectively. Uncropped image of gel is shown in Supplementary Fig. 9.

We first examined expression of *Sbno1* in mouse preimplantation embryos. Semiquantitative reverse-transcription polymerase chain reaction (RT-PCR) analyses revealed that *Sbno1* transcripts are present in both oocytes and preimplantation embryos (Fig. 1a). The expression level decreased soon after fertilization, then recovered gradually with cell division (Fig. 1a). In contrast, Sbno1 protein was not detected in the oocyte (Fig. 1b). The first nuclear localization of Sbno1 was detected at low levels in the zygote (Fig. 1b). Robust levels of Sbno1 were observed in the nuclei of preimplantation embryos from the two-cell stage, and this nuclear localization was maintained during cell division and compaction (Fig. 1b). At embryonic day 3.5 (E3.5) the embryo has developed into a blastocyst, which consists of the ICM, outer TE and blastocoel. Sbno1 was detected in the nuclei of both ICM and TE cells (Fig. 1b). Throughout the developmental processes, Sbno1 was observed exclusively in the nucleus, suggesting a nuclear function. Expression patterns of *Sbno1* at later stages are shown in Supplementary Fig. 1.

We then generated *Sbno1* knockout mice by targeting exon 7 of *Sbno1*, which harbors the N-terminal DExH box region (Supplementary Figs 2 and 3). After Cre-mediated recombination, the targeted allele contains a frame-shift, resulting in a premature stop codon. We could not obtain *Sbno1* homozygous embryos (*Sbno1*^Δ/Δ^) from *Sbno1*^Δ/+^ intercrosses at post-implantation stages, indicating embryonic lethality during very early stages of development. To examine further, we collected preimplantation embryos. At E3.5, control heterozygous embryos developed to the blastocyst stage. In contrast, *Sbno1* knockout embryos did not form the blastocoel but retained an un-compacted morphology (Fig. 1c,d). When Sbno1 protein was checked by immunohistochemistry, it was absent from the two-cell stage in *Sbno1* knockout embryos (Fig. 1f), whereas the signal was clearly evident in *Sbno1*^Δ/+^ embryos from E1.5 to E3.5 (Fig. 1e), confirming the absence of Sbno1 in the knockouts.

Next, we performed *in vitro* embryo culture to observe serial development of *Sbno1* knockout embryos from the two-cell to blastocyst stage (Fig. 1g,h, Supplementary movie S1). *Sbno1* knockout embryos compacted normally at the morula stage but failed to form the blastocoel, resulting in fragmentation of the embryo 72 hours after initiation of *in vitro* culture. Extensive cell death then occurred, as shown by TUNEL staining (Fig. 1j). Under these culture conditions, control *Sbno1*^Δ/+^ embryos gave rise to blastocysts, via compaction and formation of the blastocoel (Fig. 1g), and did not show any TUNEL staining (Fig. 1i). In addition, cell proliferation was significantly repressed in the *Sbno1* knockout embryos at E3.5, as shown by phospho-Histone H3 staining (Fig. 1k,l). Quantitatively, the phospho-Histone H3 staining was reduced to approximately 20% of that in the control embryos (Fig. 1m). These results indicate that Sbno1 regulates a critical step of the morula-to-blastocyst transition, and that a loss of Sbno1 function results in cell cycle arrest and consequent apoptotic cell death after the 16-cell stage. Consistent with this cell death in the *Sbno1* knockouts, *Sbno1* knockout embryonic stem (ES) cells could not increase cell number, and intensive cell death occurred (Supplementary Fig. 4), indicating that Sbno1 is essential for cellular survival of ES cells.

### Trophectoderm markers are not induced in *Sbno1* knockout embryos

**Figure 2:**
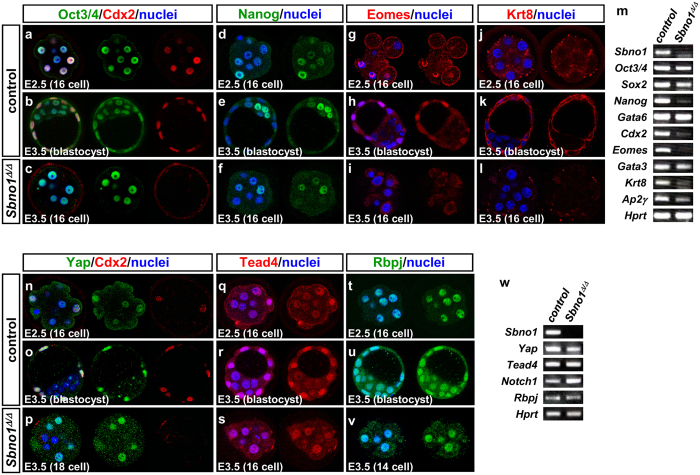
Expression of markers for the inner cell mass (ICM) and trophectoderm (TE), and for Yap/Tead4 and Notch1/Rbpj in control and *Sbno1*^Δ/Δ^ embryos. (**a**–**l**) Immunohistochemistry of ICM (Oct3/4, Nanog) and TE markers (Cdx2, Eomes; Eomesodermin, Krt8; Keratin 8) in E2.5/E3.5 control (**a**,**b**,**d**,**e**,**g**,**h**,**j**,**k**) and E3.5 *Sbno1*^Δ/Δ^ embryos (**c**,**f**,**i**,**l**). In 16-cell *Sbno1*^Δ/Δ^ embryos at E3.5, Oct3/4 and Nanog were expressed at similar levels to those in the 16-cell stage control embryos (**a**–**f**). On the other hand, expression of Cdx2, Eomes and Krt8 was not evident in *Sbno1*^Δ/Δ^ embryos (**a**–**c**,**g**–**l**). (**m**) RT-PCR analysis of E3.5 control and *Sbno1*^Δ/Δ^ embryos. Although *Oct3/4, Sox2* and *Gata6* were expressed at normal levels, expression of *Nanog, Cdx2, Eomes* and *Krt8* was greatly decreased in *Sbno1*^Δ/Δ^ embryos. *Hprt* was used as an internal control. (**n**–**v**) Immunohistochemistry of Yap, Cdx2, Tead4 and Rbpj in E2.5/E3.5 control (**n**,**o**,**q**,**r**,**t**,**u**) and E3.5 *Sbno1*^Δ/Δ^ embryos (**p**,**s**,**v**). Nuclear localization of Yap in the Cdx2-expressing TE cells was observed in the control embryos (**n**,**o**), In *Sbno1*^Δ/Δ^ embryos, expression of Cdx2 was very faint but Yap is clearly localized in nuclei (**p**). Expression of Tead4 and Rbpj in *Sbno1*^Δ/Δ^ embryos was as the same as that in the control (**q**–**s**,**t**-**v**). Semiquantitative RT-PCR analysis showed expression of *Yap, Tead4, Notch1* and *Rbpj* in control and *Sbno1*^Δ/Δ^ embryos at E3.5 (**w**). Uncropped image of gels are shown in Supplementary Fig. 10.

To investigate whether Sbno1 might regulate the expression of genes that are essential for the morula-to-blastocyst transition, we examined expression of genes that mark the differentiation of the ICM and TE in E2.5 control (wild-type), E3.5 control (*Sbno1*^+/+^ or *Sbno1*^Δ/+^) and *Sbno1* knockout embryos. One of the ICM markers, Oct3/4, was observed equally in both the control and *Sbno1* knockout embryos at E2.5–E3.5 (16-cell stage), as revealed by immunohistochemistry (Fig. 2a–c). Again, the *Sbno1* knockouts did not develop to the blastocyst stage, yet Oct3/4 levels were maintained, even at E3.5 (Fig. 2c). At E3.5, Nanog levels remained low in the *Sbno1* knockouts (Fig. 2f), at the same level as the control at E2.5 (Fig. 2d), whereas they were increased in the ICM of the control at E3.5 (Fig. 2e), indicating that reduced levels of Nanog in the *Sbno1* knockouts were due to developmental delay. Consistent with the immunohistochemistry, the semiquantitative RT-PCR analysis with E3.5 embryos revealed that three ICM markers, *Oct3/4, Sox2* and *Gata6*, were expressed at the same level in both controls and knockouts, yet expression of *Nanog* was decreased solely in the *Sbno1* knockouts (Fig. 2m). These data indicate that formation of the ICM was unaffected in the absence of Sbno1, except for the decline of Nanog at E3.5.

In contrast to the ICM markers, Cdx2, an early TE marker, was barely detectable in the *Sbno1* knockout embryos at E2.5 and E3.5 (Fig. 2c, Supplementary Fig. 5), whereas Cdx2 was evident in both E2.5 embryos and the TE cells in the control blastocysts at E3.5 (Fig. 2a,b, Supplementary Fig. 5). Although the development of *Sbno1* knockout embryos was slightly delayed at E2.5, Cdx2 expression was not turned on even in the 16-cell *Sbno1* knockout embryos at E3.5, suggesting that induction of Cdx2 expression is not normal in the absence of Sbno1. Other TE markers, such as Eomesodermin (Eomes) and Keratin 8 (Krt8), whose expression is regulated by Cdx2^21^,^22^, were also absent in the *Sbno1* knockout embryos at E3.5 (Fig. 2g–m). The absence of or reduced expression of *Cdx2, Eomes* and *Krt8* was further confirmed by semiquantitative RT-PCR analysis at E3.5 (Fig. 2m). These results indicate that differentiation of the TE is severely impaired in the absence of Sbno1.

Yap and Tead4 interact in the nuclei of the outer cells of the preimplantation embryo^3^, acting upstream in TE differentiation. In cooperation with Yap/Tead4, Notch/Rbpj signaling directs TE fate by regulating *Cdx2* transcription^7^. Hence, we checked protein levels of Yap, Tead4 and Rbpj, and found that these essential components were expressed normally with correct nuclear localization (Fig. 2n–w). This strongly suggests that Yap/Tead4 and Notch/Rbpj could not activate *Cdx2* in the absence of Sbno1, even though they were correctly localized in the nucleus. This suggests a regulatory role of Sbno1 on the Yap/Tead4 and Notch/Rbpj-mediated transcription of *Cdx2*. Sbno1 is a nuclear protein; therefore, we characterized its molecular functions as a novel transcriptional regulator.

### Sbno1 stimulates Yap/Tead and Notch/Rbpj transcriptional activities

**Figure 3:**
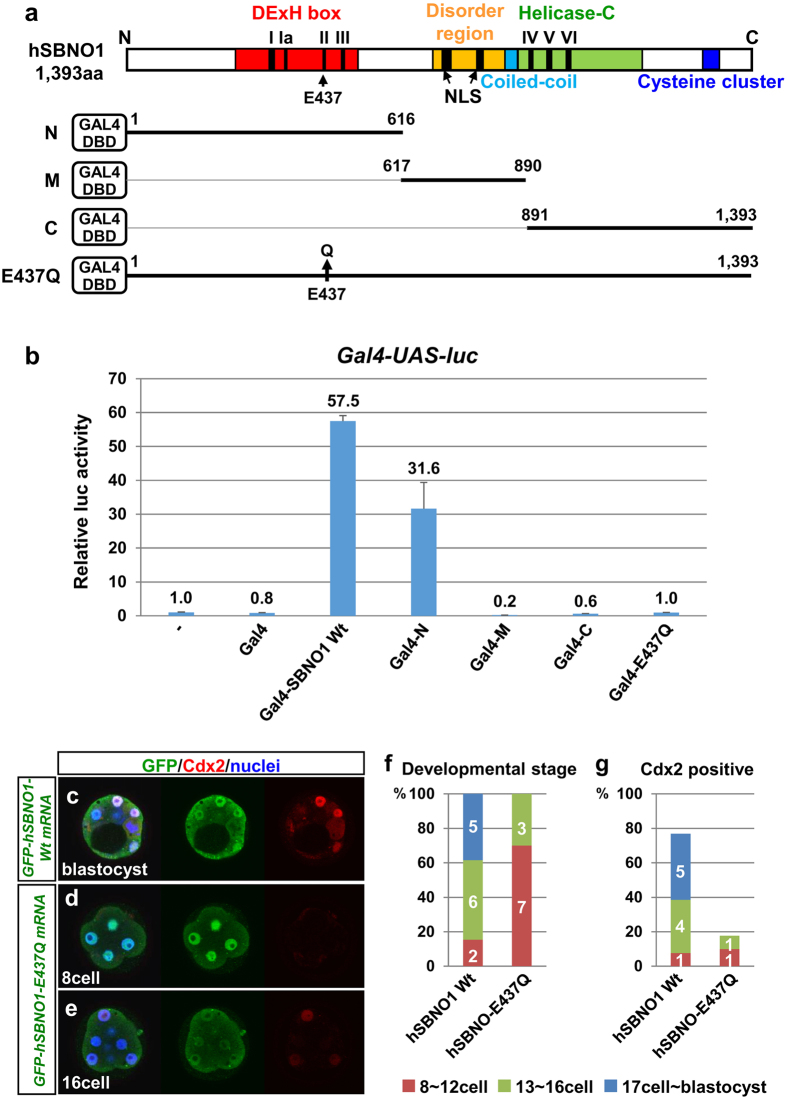
Effects of human SBNO1 and SBNO1 mutants on transcription and preimplantation development. (**a**) DExH box, disorder region with two nuclear localization signals (NLS), coiled-coil, helicase-C domains and cysteine cluster in human SBNO1 are indicated. Seven conserved motifs (I to VI) were found in the DExH box and helicase-C domains. Deletion and E437Q mutants of hSBNO1 used in Gal4-luciferase assays are shown. (**b**) Transcriptional activation profiles of the full length, deletion and E437Q mutants were obtained by Gal4-luciferase assays in 293 T cells. Gal4-hSBNO1 acted as a robust transcriptional activator. The N-terminal region of hSBNO1 retained its activator function, whereas the middle and C-terminal regions did not convey activity and produced a repressive effect. Note that the E437Q mutant completely lost activity on transcription. All data are presented as means ± SD. (c-g) mRNA injection of GFP-hSBNO1-E437Q to zygotes resulted in developmental arrest and Cdx2 reduction after 3days culture, on the other hand, embryos showed little effect by GFP-hSBNO1-Wt mRNA injection.

Careful inspection of the protein structure of Sbno1 (human, mouse, fly and nematode) and comparison with other factors revealed several key features of Sbno1 as a member of the SF2 family, which includes DExD/H box helicases and Swi2/Snf2 remodelers (Fig. 3a, Supplementary Fig. 3)^23^,^24^,^25^,^26^. Sbno1 proteins are highly conserved among species (human vs mouse 97.6%, vs fly 55.3%, vs nematode 47.4%), and two characteristic features of the SF2 family, namely the DExH box and helicase-c domains, are found in the N- and C-terminal regions of Sbno1, respectively (Supplementary Fig. 3). These distinctive features strongly suggest that Sbno1 might be involved in transcriptional control of gene expression by acting as a chromatin remodeler.

In addition to the structural aspect, genetic analyses of *strawberry notch (sno*), a *Drosophila* ortholog of *Sbno1*, suggest that sno positively regulates transcription of its targets to activate Notch (*Delta*), Wnt (*wingless*) and Hippo (*scalloped, vestigial*) signaling pathways^12^,^13^,^18^,^27^,^28^. These lines of evidence indicate that Sbno1/sno might be a crucial transcriptional integrator acting at the intersection of different signaling cascades.

To investigate this possibility, we first made an artificial construct, in which human *SBNO1* was fused with a gene encoding the Gal4-DNA binding domain. This binds to Gal4-binding sequence multimerized and inserted upstream of the chicken *δ-crystalline* minimal promoter and the luciferase reporter^29^. When the Gal4-luciferase assay was performed, Gal4-SBNO1 induced robust activation of transcription (Gal4-SBNO1 Wt, 57.5-fold; Fig. 3b), whereas the Gal4-DNA binding domain alone did not show any effect on luciferase activity (Gal4, 0.8-fold; Fig. 3b), indicating that Sbno1 acts as a potent transcriptional activator. Next, we constructed deletion mutants, by dividing SBNO1 into three regions (N-terminal, Middle and C-terminal parts; Fig. 3a). Gal4 fused with the SBNO1-N terminal region (Gal4-N) retained the luciferase activity, whereas the activator function was lost when the M and C regions were used (Fig. 3b). The N-terminal region harbors the DExH box containing ATPase activity; therefore, we mutated E437, an essential glutamic acid residue for DExH domain ATPase, activity^30^,^31^ to glutamine (Q) (Fig. 3a). As expected from the essential role of ATPase activity in the SF2 family^30^,^31^, the Gal4-SBNO1-E437Q mutant completely lost luciferase activity (Fig. 3b).

To address whether the Sbno1 activity is required for the preimplantation development, we microinjected *EGFP-hSBNO1-Wt* or -*E437Q* mutant mRNA to mouse zygotes, and cultured them for 3 days. Contrary to *hSBNO1-Wt*, development of most of *hSBNO1-E437Q*-injected embryos arrested at 8–12 cell stages (Fig. 3c–f), earlier than the *Sbno1* knockouts (Figs 1 and 2), and this arrest was accompanied by reduction of Cdx2 expression (Fig. 3g). This result indicates that E437 in the DExH domain is crucial for the function of Sbno1 during preimplantation development.

**Figure 4:**
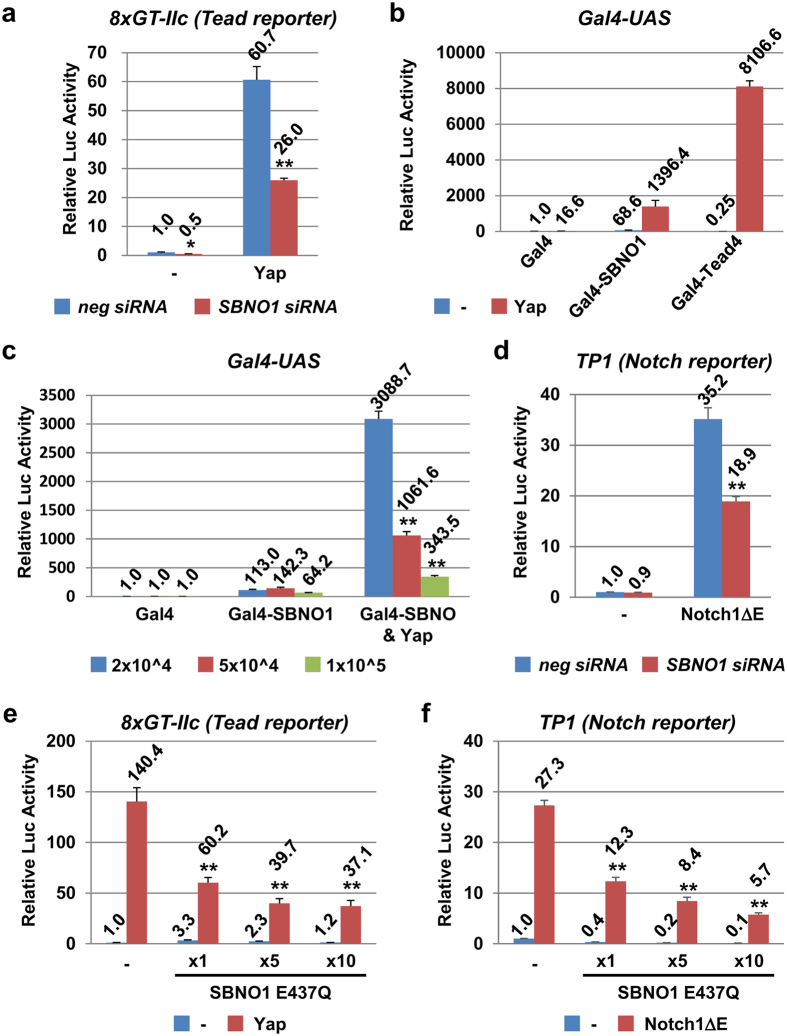
Contribution of Sbno1 to transcriptional activation mediated by Yap/Tead and Notch/Rbpj. (**a**) Transcriptional activities of δ-crystalline minimal promoter-luciferase with Tead binding sites (8xGT-IIc Tead reporter) were measured. Yap activated this Tead reporter (60.7-fold), and the activation was repressed by knocking down endogenous SBNO1 (*SBNO1* siRNA) (26.0-fold). (**b**) Transcriptional activities of δ-crystalline minimal promoter-luciferase with Gal4-binding sites (Gal4-UAS) were measured. Gal4-SBNO1 alone stimulated transcription (68.6-fold), and this activation was further enhanced by Yap (1396.4-fold). GAL4-Tead4, which did not activate the reporter alone (0.25-fold), showed robust synergistic activation with Yap (8106.6-fold). (**c**) When 293 T cells were seeded at different cell densities (2 × 10^4^, 5 × 10^4^ and 1 × 10^5^ cells/well in 24-well plates the synergistic activation by Gal4-SBNO1 and Yap was robust at the low cell density (3088.7-fold), but not at the high cell density (343.5-fold). (**d**) The TP1 Notch reporter (12x Rbpj binding sites-βglobin promoter-Luc) was activated by Notch1ΔE (35.2-fold), and this activation was repressed by *SBNO1* siRNA (18.9-fold). (**e**,**f**) Activation of the 8xGT-IIc Tead reporter by Yap (**e**) and TP1 Notch reporter by NotchΔE (**f**) was reversed by the SBNO1-E437Q mutant in a dose-dependent manner. All data are presented as means ± SD. **p < 0.01 versus relevant control.

Expression of *Cdx2* was dramatically reduced in the *Sbno1* null embryos; therefore, we speculate that Sbno1 might be involved in the transcriptional control by the Yap/Tead4 and Notch/Rbpj complexes, which act through the Hippo and Notch cascades, respectively, to directly regulate *Cdx2*^7^. Importantly, *Drosophila* sno functions on these two signaling networks^18^. To explore this possibility, we analyzed transcriptional control by the Yap/Tead complex, using a reporter that contains eight repeats of the Tead binding site (5′-GCTGTGGAATGTGTGTC-3′) upstream of a minimal *δ-crystallin* promoter (8xGT-IIc-Luciferase)^32^. When 293 T cells were transfected with this reporter, along with a Yap expression plasmid, robust activation of the luciferase reporter was observed (60.7-fold; Fig. 4a). This activation was repressed by endogenous *SBNO1* knockdown (26.0-fold; Fig. 4a), but the reporter without Tead binding sites was unaffected (data not shown). Reduction of SBNO1 protein levels by siRNA was confirmed by western blotting using an anti-SBNO1 antibody (Supplementary Fig. 6). These results indicate a contribution of Sbno1 to Yap/Tead-mediated transcriptional activation.

To explore further the function of Sbno1 in Yap/Tead-mediated Hippo signaling, we determined the effects of Gal4-SBNO1 or Gal4-Tead4-mediated transcriptional activation on Yap (Fig. 4b). As expected, in the presence of Yap, the Gal4-Tead4 fusion protein activated the Gal4 reporter robustly, by approximately 8,100-fold, confirming the reliability of this assay. Gal4-SBNO1 alone activated the reporter (68.6-fold), but when Yap was co-expressed, enhancement of activation was observed (approximately 1,400-fold; Fig. 4b).

Yap nuclear localization is promoted by escaping cell contact inhibition^32^,^33^; therefore, we performed the same experiment with different cell densities (Fig. 4c). Gal4-SBNO1 alone exhibited similar effects, regardless of cell density (113-, 142- and 64-fold activation). In clear contrast to this, when Yap was co-expressed, super-activation of Gal4-SBNO1 (3088-fold) was observed at low cell density, yet this robust activation was largely repressed at high cell density to 343-fold. This strongly suggests that Sbno1 can act in concert with the Hippo pathway, which is known as a sensor of the physical milieu, as represented by cell density^32^,^34^,^35^.

Involvement of Sbno1 on Notch/Rbpj transcriptional activity was also examined with a *TP1-luciferase* reporter, which contains 12 copies of the Rbpj binding site, and is activated by Notch intracellular domain (NICD) and Rbpj^36^. When luciferase activities derived from this reporter were measured in 293 T cells, the reporter was activated by an active form of Notch1 (Notch1ΔE, extracellular domain-deleted)^37^, and this transcriptional activation was repressed by co-introduction of *SBNO1* siRNA (Fig. 4d), indicating the requirement of Sbno1 for efficient transcription of Notch/Rbpj target genes.

The hSBNO1-E437Q mutant lacks function as a transcriptional activator (Fig. 3b); therefore, we speculated that this mutant SBNO1 would act as a dominant negative mutant. To confirm this possibility, we expressed hSBNO1-E437Q along with the Tead or Notch reporter and their effectors, Yap and Notch1ΔE, respectively. Consistent with the *SBNO1* siRNA experiments, hSBNO1-E437Q repressed both the Tead and Notch reporters in a dose-dependent manner (Fig. 4e,f). These results revealed that intact Sbno1 DExH box activity is indispensable for the transcriptional activation of Tead and Rbpj by their co-activators, Yap and Notch, respectively.

### Sbno1 is necessary for normal *Cdx2* trophectoderm enhancer activity

**Figure 5:**
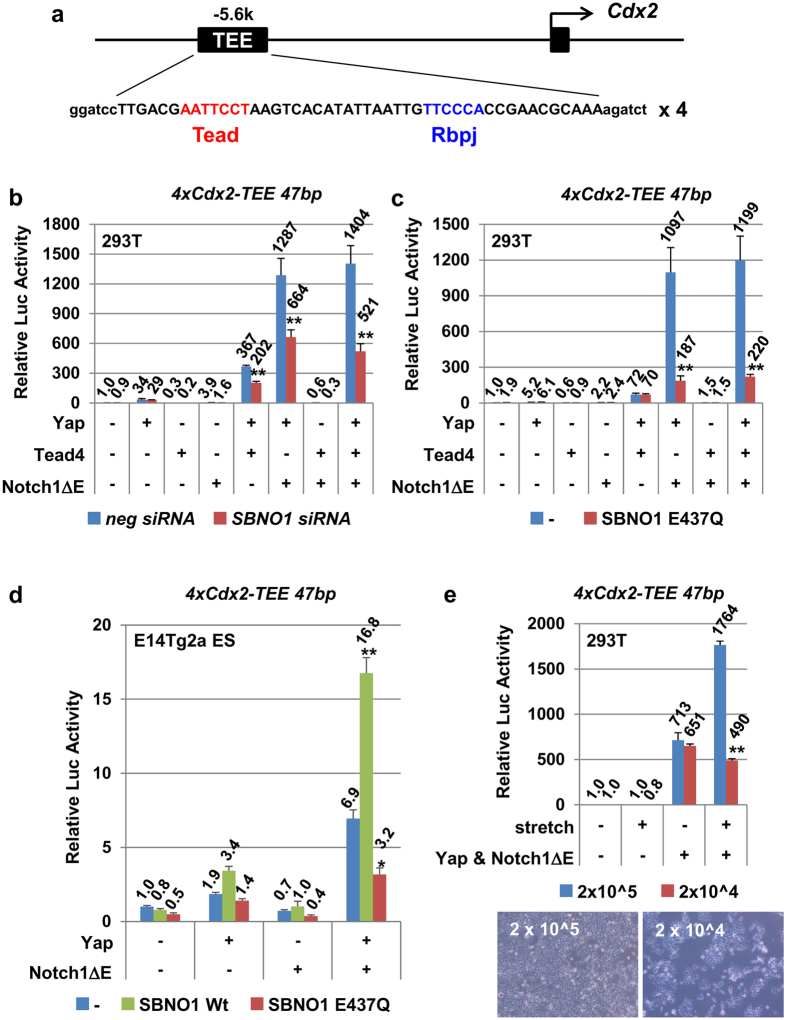
Involvement of Sbno1 in transcriptional activation of the *Cdx2* trophectoderm enhancer (*Cdx2*-TEE) in 293 T and E14Tg2a ES cells. (**a**) Location and core sequences of the *Cdx2*-TEE are shown. Tetramerized 47 bp *Cdx2*-TEE was ligated to δ-crystalline minimal promoter-luciferase (*4xCdx2-TEE47bp*). (**b**) Expression of Yap alone or NotchΔE alone activated the *4xCdx2-TEE47bp* reporter (39- and 3.9-fold, respectively), yet when both Yap and NotchΔE were co-expressed, this reporter was synergistically and robustly activated (1287-fold). As expected, this activation was repressed by *SBNO1* siRNA (664-fold). Synergism between Yap and Tead4 was observed (367-fold); however, expression of Tead4 did not affect the synergistic activation by Yap and NotchΔE (1287- versus 1404-fold activation). (**c**) Lack of transcriptional activation of the *4xCdx2-TEE47bp* reporter was evident when the SBNO1-E437Q mutant was expressed. (**d**) Transcriptional activity of the *4xCdx2-TEE47bp* reporter was synergistically upregulated by Yap and NotchΔE in E14Tg2a ES cells, and the SBNO1-E437Q mutant significantly decreased the activity (6.9- and 3.2 fold, respectively). In contrast, SBNO1-Wt increased the Yap and NotchΔE-induced transcriptional activity (16.8 fold). (**e**) Synergistic activation of the *4xCdx2-TEE47bp* reporter by Yap and NotchΔE was observed at both high and low cell densities in the absence of mechanical stretch (713- and 651-fold, respectively). In contrast, when cells were stretched, this synergistic activation was super-enhanced to 1764-fold only in the high cell density culture. At low cell density, mechanical enhancement of transactivation was not observed, and was slightly repressed (490-fold). Pictures of cell cultures are shown. Note that cells make mutual contacts at high density, while at low density cells are isolated or clustered in small separated islands of cells. All data are presented as means ± SD. **p < 0.01 versus relevant control.

In *Sbno1* knockout embryos, Cdx2 expression was significantly decreased (Fig. 2). Recently, expression of *Cdx2* in the TE has been shown to be regulated by Yap/Tead4 and Notch/Rbpj though binding to sites in the trophectoderm-enhancer (TEE) in the *Cdx2* gene^7^. To analyze the roles of Sbno1, we made a new luciferase reporter, in which a short element (47 bp) of the *Cdx2* TEE containing both the Tead and Rbpj biding sites was tetramerized and inserted in front of the minimal *δ-crystallin* promoter (*4xCdx2-TEE47bp*; Fig. 5a). As expected, Yap and Tead4 synergistically activated *4xCdx2-TEE47bp* in 293 T cells (367-fold; Fig. 5b), whereas sole expression of Yap or Tead4 resulted in only mild activation or repression (34- and 0.3-fold, respectively; Fig. 5b). Notch1ΔE alone produced weak activation (3.9-fold, respectively; Fig. 5b). Nonetheless, when Yap and Notch1ΔE or all three effectors were simultaneously introduced, activation of *4xCdx2-TEE47bp* increased by 1,287 and 1,404-fold, respectively (Fig. 5b). The activation of *Cdx2*-TEE was significantly suppressed by knocking-down *SBNO1* (Fig. 5b). When the hSBNO1-E437Q mutant was used instead of knock-down, repression of synergistic activation by Yap, Tead4 and Notch1ΔE was more evident, resulting in approximately 20% activation (Fig. 5c).

Similarly to 293 T cells, E14Tg2a ES cells showed synergistic activation of the *4xCdx2-TEE47bp* by Yap and Notch1ΔE. This synergism was again significantly suppressed by the hSBNO1-E437Q mutant, whereas the hSBNO1-Wt enhanced the cooperative effect of Yap and Notch1ΔE on the *4xCdx2-TEE47bp* in E14Tg2a ES cells (Fig. 5e), although it did not influence to the activities of *4xCdx2-TEE47bp* reporter in 293 T cells (Supplementary Fig. 7). These results clearly demonstrate that Sbno1 is an essential component at the convergence of two different signaling cascades, namely Hippo and Notch.

As shown in Fig. 4c, transcriptional activation by Yap is dependent on cell density. Recently, the Hippo cascade has been shown to be sensitive to cytoskeletal tension, highlighting Yap as a mechanotransducer^34^,^35^,^38^,^39^,^40^,^41^,^42^. Likewise, physical force can activate the Notch signaling^43^. Hence, the Notch and Hippo cascades are both sensitive to the physical state of cells. Next, we confirmed whether activation by these two pathways is also sensitive to physical parameters (Fig. 5e) by stretching transfected cells on a silicone membrane. Even at high or low cell density, co-transfection of Yap and Notch1ΔE activated *4xCdx2-TEE47bp* robustly, and at the same intensity (713 vs. 651-fold induction). More importantly, the transactivation was super-enhanced when cells were stretched at high cell density (a 713–1764-fold induction), whereas the mechanical stretch at a low cell density had a repressive effect (651–490-fold). These lines of evidence indicate that transcriptional activation of *Cdx2* through the TEE is dependent on the physical state of cells.

### Sbno1 physically interacts with Yap/Tead4 and NICD1/Rbpj

**Figure 6:**
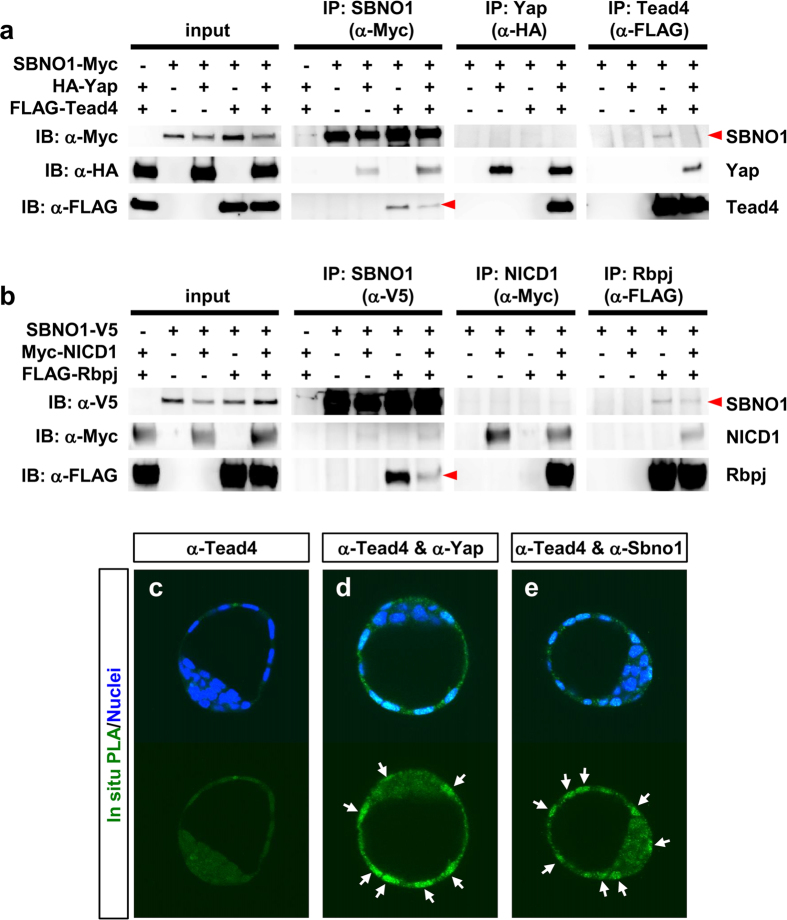
Physical interaction between SBNO1 and Yap/Tead4 or Notch/Rbpj. (**a**) SBNO1-Myc, HA-Yap, and/or FLAG-Tead4 were co-expressed in 293 T cells, and immunoprecipitated with indicated antibodies (α-Myc, α-HA and α-FLAG, respectively). Interactions between HA-Yap and FLAG-Tead4, and SBNO1-Myc and FLAG-Tead4 were observed by co-precipitation. Expression of HA-Yap attenuated the interaction between SBNO1-Myc and FLAG-Tead4, as observed by faint bands of co-precipitation (red arrowheads). Myc-SBNO1 co-precipitated HA-Yap, whereas HA-Yap did not co-precipitate SBNO1-Myc. (**b**) SBNO1-V5, Myc-NICD1, and/or FLAG-Rbpj were co-expressed in 293 T cells, and immunoprecipitated with indicated antibodies (α-V5, α-Myc and α-FLAG, respectively). FLAG-Rbpj was co-immunoprecipitated with SBNO1-V5 (α-V5) and Myc-NICD1 (α-Myc), indicating their interaction. This was further confirmed by co-precipitation of SBNO1-V5 and Myc-NICD1 with FLAG-Rbpj (α-FLAG). The interaction between SBNO1 and Rbpj was weak in the presence of NICD1, as observed by faint bands (red arrowheads). (**c**–**e**) Interaction between Sbno1 or Yap and Tead4 in E3.5 embryos was analyzed by *in situ* PLA. Green signals indicate the ligated antibodies, which represent physical interaction of the antigens. Antibodies against Sbno1 or Yap with Tead4 gave clear signals in the nuclei of outer cells (**d**,**e**), while α-Tead4 antibody alone did not give any signal (**c**). Uncropped image of blots are shown in Supplementary Fig. 11.

Cooperative regulation of *Cdx2* by Sbno1, Yap/Tead4 and Notch/Rbpj suggests that these factors physically interact with each other. To explore this, we carried out co-immunoprecipitation (CoIP) analysis, and found that SBNO1 indeed interacts physically with Tead4, as revealed by co-precipitation of SBNO1 with Tead4 as well as Yap (IP:α-Myc; Fig. 6a). In contrast, when Yap was precipitated by an anti-HA antibody (IP:α-HA; Fig. 6a), only Tead4 was co-precipitated; therefore, interaction between SBNO1 and Yap might only be detected in limited conditions. When Tead4 was precipitated, co-purification of SBNO1 was observed weakly only in the absence of Yap (IP:α-FLAG; Fig. 6a). These data suggest that the majority of Tead4 and Yap forms a complex, and that only a fraction of this complex co-exists with SBNO1. In addition, these data also suggest that interaction between Tead4 and SBNO1 becomes weak in the presence of Yap, despite formation of the Tead4 and Yap complex.

We confirmed the interaction between SBNO1 and Rbpj, which was previously reported in *Drosophila* and zebrafish^13^,^19^. When SBNO1 was precipitated, both Rbpj and NICD were co-purified, albeit NICD1 co-precipitation was very weak (Fig. 6b). When NICD1 was precipitated, only Rbpj was co-purified (Fig. 6b). Likewise, SBNO1 and NICD were co-precipitated along with Rbpj (Fig. 6b). More importantly, however, interaction of SBNO1 with Rbpj was again attenuated by NICD1, as represented by fainter bands of SBNO1 and Rbpj (Fig. 6b), a similar observation to that of Yap/Tead4 (Fig. 6a). These observations suggest that interaction of SBNO1 with DNA-binding proteins, such as Tead4 and Rbpj, weaken when their co-activators (Yap and NICD1, respectively) arrive in the nucleus and bind to their partners.

We next confirmed the physical interaction of Sbno1 and Tead4 in E3.5 embryos by *in situ* proximity ligation assay (PLA). Consistent with the *in vitro* CoIP analyses, the PLA signal between Sbno1 and Tead4 proteins was observed in the outer cells where Cdx2 is expressed. As expected, the Yap and Tead4 interaction gave the signal at the same level (Fig. 6c–e), indicating that Sbno1 and Yap/Tead4 make a complex in the TE cells.

### Sbno1 physically interacts with FACT (FAcilitates Chromatin Transcription), a histone chaperone for transcription

To gain more insight into the role of Sbno1 in the transcriptional control of *Cdx2*, we mined a previous high-throughput interactome analysis in HeLa cells^44^, and found SSRP1 and Cxorf26 as interacting partners for SBNO1. Although the function of Cxorf26 is unknown, Ssrp1, a high mobility group (HMG) domain-containing protein, is known to heterodimerize with Spt16 to form the FACT complex. This complex acts as a histone H2A/H2B chaperon to assist progression of RNA polymerase II on its DNA template during transcriptional elongation^45^,^46^, thereby positively controlling gene expression.

**Figure 7:**
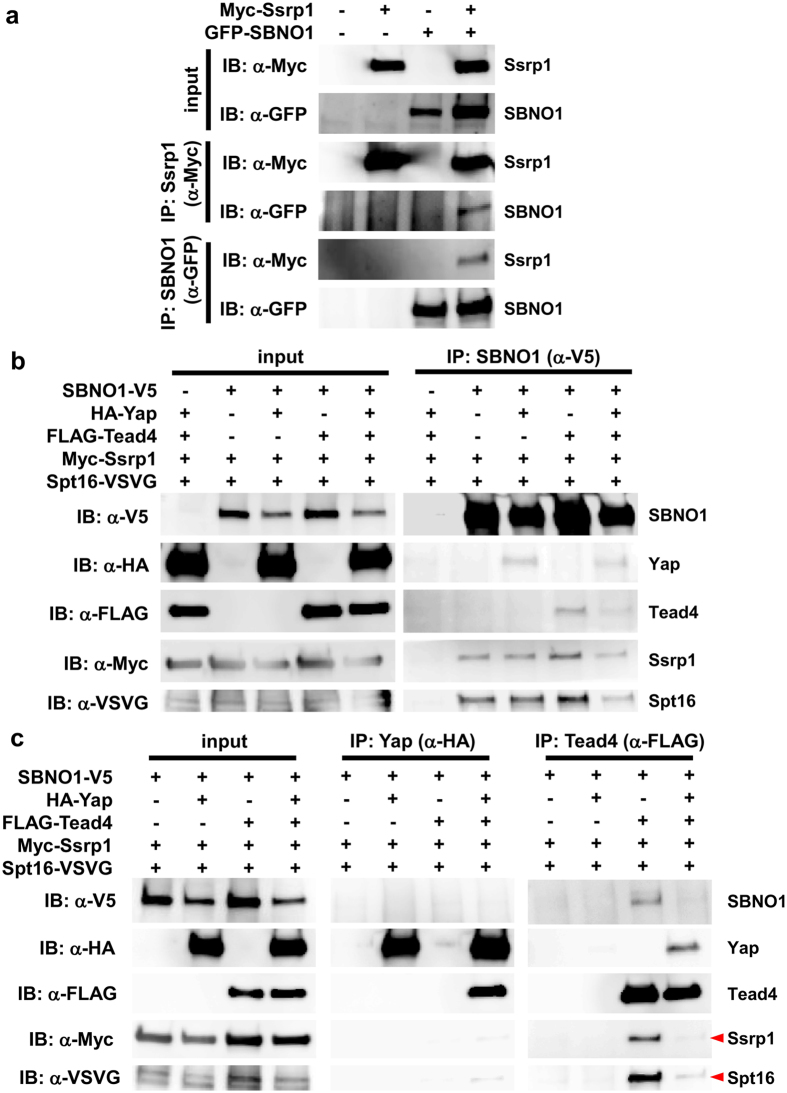
Physical interaction between SBNO1 and the FACT complex. (**a**) Interaction between SBNO1 and Ssrp1 was analyzed by co-immunoprecipitation. GFP-SBNO1 and Myc-Ssrp1 were expressed in 293 T cells and immunoprecipitated with indicated antibodies (α-GFP and α-Myc, respectively). GFP-SBNO1 was co-precipitated along Myc-Ssrp1 and *vice versa*. (**b**) SBNO1-V5, HA-Yap, FLAG-Tead4, Myc-Ssrp1 and/or Spt16-VSVG were co-expressed in 293 T cells. Yap, Tead4, Ssrp1 and Spt16 were co-precipitated with SBNO1, indicating formation of a complex. (**c**) When HA-Yap was precipitated, co-purified bands of Myc-Ssrp1 and Spt16-VSVG were faint (α-HA). Both Myc-Ssrp1 and Spt16-VSVG were co-precipitated with FLAG-Tead4, yet in the presence of HA-Yap, bands corresponding to Myc-Ssrp1 and Spt16-VSVG became faint (red arrowheads), indicating weak interaction between Tead4 and the FACT complex (α-FLAG). Uncropped image of blots are shown in Supplementary Fig. 12.

To determine whether SBNO1 could be a crucial component of this chaperone machinery, we examined the interaction between SBNO1 and Ssrp1 using the CoIP assay. When SBNO1 or Ssrp1 was precipitated, Ssrp1 and SBNO1 were co-purified, respectively (Fig. 7a). We then further probed the relationship between SBNO1 and the FACT complex in the presence or absence of Yap and Tead4. In both cases, Ssrp1 and Spt16 were co-precipitated along with SBNO1 (Fig. 7b), although again precipitation of Tead4 became inefficient in the presence of Yap, as observed in Fig. 6a. When Yap was precipitated, no Ssrp1 or Spt16 was co-purified (IP:α-HA; Fig. 7c), indicating a weak interaction between Yap and the FACT complex. In contrast, Tead4 interacted strongly with Spt16 and Ssrp1, although this interaction became weak when Yap was present (IP:α-FLAG; Fig. 7c). These lines of evidence suggest that the FACT factors make a complex with SBNO1 and Tead4, yet formation of this complex is transient. When co-activators, such as Yap, are recruited to the complex, Spt16 and Ssrp1 are released, which may be a mechanism to control their histone chaperone activity to facilitate transcriptional elongation of target genes.

## Discussion

Our analysis clarifies a pivotal role of Sbno1 in preimplantation development. The transcriptional level of a key TE determinant, *Cdx2*, is stimulated by Sbno1 enzymatic activity, along with Yap/Tead4 and Notch/Rbpj transcriptional complexes. Physical interaction between Sbno1, Yap/Tead, Notch/Rbpj and FACT complexes indicates that Sbno1 coordinates association of DNA, transcription factors and histones. These findings describe a critical function of a helicase-related factor on gene transcription during cellular differentiation.

During development of mouse preimplantation embryos, genes encoding essential components of the Notch signaling pathway are expressed^47^. Nonetheless, Notch signaling seemed to be dispensable, because maternal/zygotic knockout embryos of *Rbpj, Notch1*, or *O-fucosyltransferase 1* can implant normally and survive until E9.5^48^,^49^. Contrary to these observations, Rayon *et al*., reported Notch/Rbpj activity in TE cells and, more importantly, that expression of *Cdx2* in TE cells is regulated by both Notch/Rbpj and Yap/Tead4 through their direct binding to the TEE^7^. Our study also shows that Notch/Rbpj and Yap/Tead4 synergistically activate the *Cdx2*-TEE in both HEK293 and ES cells, and this synergism was interrupted by the hSBNO1-E437Q mutant, which is deficient in ATPase activity (Fig. 5). The synergistic activation on the *Cdx2*-TEE was robust when Yap and Tead4 are used in our assay, a clear contrast to Tead4-VP16, which gave only 3-fold activation^7^. This could be a difference between reporter constructs, since we multimerized the *Cdx2*-TEE elements in our luciferase reporter. As another possibility, an artificial fusion activator Tead4-VP16 could not interact with Yap and/or NICD normally, failing to achieve the physiological activation, which requires Sbno1. Nonetheless, both reports clearly show that the Notch and Yap cascades synergistically activate the *Cdx2*-TEE, with Sbno1 acting as a signal integrator of these two different cascades.

In addition to the TEE region, Tead4 binds to several genomic sites on the *Cdx2* locus in blastocysts and trophoblast stem cells^50^, and combination of different regulatory elements might be necessary for the robust Cdx2 expression in the TE^51^. Interestingly, *Tead4* knockout embryos can form blastocoel with Cdx2 and other TE gene expression when cultured under a hypoxic condition^52^, and it is intriguing whether regulatory roles of Sbno1 on transcriptional activation and cellular survival might depend on oxygen concentration.

Although Sbno1 expression is ubiquitous in preimplantation embryos and at later developmental stages (Supplementary Fig. 1), it can regulate distinct target genes, namely *Cdx2*, in a temporally and spatially controlled manner via interactions with Yap/Tead4 and Rbpj in the TE of preimplantation embryos. In post-implantation embryos, Sbno1 might control development of the neural tube and presomitic mesoderm, where its expression is evident (Supplementary Fig. 1). Importantly, these two tissues require Notch signaling for their proper development^53^,^54^. Moreover, Hippo signaling is involved in the control of the size of the neural progenitor pool^55^,^56^. Although Sbno1 function in the presomitic mesoderm is not known, these data strongly suggest that the same mechanism functions during neural development. Recently, it has been reported that the Notch and Hippo cascades regulate homeostasis of crypts in the intestinal epithelium^57^,^58^, in which *Cdx2* is expressed^59^. Analyses should be expanded to other organs and their cancers, such liver/hepatocarcinoma, colon/colorectal cancers and pancreas/pancreatic cancers, because the Notch and Hippo pathways play critical roles during carcinogenesis^60^,^61^,^62^.

*Sbno1* knockout embryos after E3.5 and *Sbno1* knockout ES cells showed remarkable cell death. These results indicate that Sbno1 is an essential factor, not only for the *Cdx2* transcriptional regulation but also for the cellular survival in pre- and peri-implantation embryos. FACT complex is known to regulate transcriptional elongation, but also required for DNA repair^63^. Loss of Ssrp1 in mouse embryos causes peri-implantation lethality^64^, and depletion of Ssrp1 or Spt16 in ES cells results in cell death^65^, suggesting that the function of Sbno1 on cellular viability in preimplantation embryo may be associated with functions of the FACT complex.

Based on its domain structure, we conclude that Sbno1 belongs to the DExD/H helicase sub-group of the SF2 family^26^. DExD/H helicases are proposed to be ATP-dependent RNA helicases, although several DExD/H helicases are active in other areas of RNA metabolism^23^. More importantly, several members have multiple functions as transcriptional regulators, which are independent of their RNA helicase activity. For example, DDX3, a DEAD-box RNA helicase, is a regulatory subunit of Casein Kinase 1 in the canonical Wnt signaling cascade^66^, highlighting a novel role of a DEAD-box protein as a crucial Wnt signal regulator. In this sense, our data impart a new role to Sbno1 as a transcriptional regulator bridging the Yap/Tead-Notch/Rbpj complexes and the FACT histone chaperone.

Previous reports have shown that transcriptional co-activators, such as NcoA6 or the mediator complex, are crucial for transcription of Yap target genes^67^,^68^, although functional relationship with the histone chaperone is largely unknown. We speculate that binding of Yap and NICD to their binding partners Tead4 and Rbpj might release Sbno1 and the FACT complex to facilitate nucleosome melting, which is essential for efficient transcriptional elongation (Supplementary Fig. 8). In the absence of Sbno1, the FACT complex might loose its access to *Cdx2* gene, resulting in a pause of transcriptional elongation that can be found in Yap/Tead targets^67^,^68^. We do not exclude a possibility that Sbno1 *per se* might help association of co-activators to the Yap/Tead4 and NICD/Rbpj complexes on *Cdx2*, since Gal4-Sbno1 acts as a robust transcriptional activator (Fig. 3b). To understand the mechanistic actions of Sbno1 and the FACT complex precisely, future studies must verify whether Sbno1 processes promoter melting and/or interacting domains to the transcriptional co-activators and chromatin remodelers.

Although we do not know whether Sbno1 possesses the ATP-dependent helicase activity of the SF2 family members, our analysis has shown that amino acid residue E437 in the DExH box (motif II, ATPase domain) of hSBNO1 is essential for its transcriptional control^30^,^31^. This is because the E437Q mutant only disrupts the synergism between the Hippo and Notch pathways, but does not inhibit Yap alone or Notch1ΔE alone when used to activate the reporters (Fig. 5c). Our data also suggest that artificial ATP analogs or small chemicals could be designed to abrogate the activity of the DExH box by binding to its pocket. Such chemicals would be antagonistic to the confluence of Yap/Tead and Notch signaling and may, therefore, be good candidates for anti-cancer drugs. It is also of interest that energy stress attenuates the growth-promoting effect of Yap/Tead via AMPK phosphorylation of the Hippo signaling components^69^,^70^,^71^. This suggests that ATP-analogs antagonistic to the Yap/Tead and Notch cascade might also be good candidates for anti-cancer drugs.

Recently, Hippo signaling was shown to be inhibited by cytoskeletal tension, and high tension sensed by the Ajuba protein, jub, inhibits the Hippo cascade to activate Yorkie-mediated transcription^42^. In addition, Yap was reported to act as a sensor of mechanical cues, such as stiffness of the extracellular matrix^34^ and the Notch receptor was shown to be activated by mechanical force via a mechanical allostery of its proteolytic cleavage site^72^. Hence, these two signaling cascades can be activated by mechanical stimuli and/or changes of the physical milieu. Interestingly, nuclear localization of Yap is interrupted when Rho/Rock signaling or myosin II ATPase is inhibited in preimplantation embryos, indicating that cell polarity and contractile force of the cell regulate Hippo signaling^5^,^73^,^74^,^75^. In preimplantation embryos, stochastic activation of Notch at the morula stage shifted to restricted activation in blastocyst TE cells^7^, but the mechanism of the activation is not understood. Because the TE cells that cover the surface of the preimplantation embryo have a flattened shape whereas the cells inside are round, cell division inside the embryo and subsequent expansion of the blastocoel could apply distinct physical forces to the TE and the cells inside (e.g. stretch vs. compression, respectively). This suggests that this mechanical difference could induce simultaneous activation of the Yap/Tead and Notch/Rbpj complexes, which would be integrated in a synergistic manner by Sbno1. As we have previously shown, physical forces can control gene expression during morphogenesis^76^,^77^; therefore, the functional relationship between the physical milieu and gene expression in the formation of the TE should be analyzed further, with particular regard to Yap/Tead, Notch/Rbpj and Sbno1.

## Methods

### Plasmids

*δ-crystalline* 51 bp minimal promoter (*δ51)-LucII* and *8xGT-IIc-δ51-LucII* were described in refs 29 and 32, respectively. *GAL4-UAS-δ51-LucII* contains four copies of the Gal4 binding site. Mouse *4xCdx2 TE enhancer (TEE) 47bp-δ51-LucII* was constructed by the insertion of four copies of the following fragment containing Tead and Rbpj binding sites (5′-ggatccTTGACGAATTCCTAAGTCACATATTAATTGTTCCCACCGAACGCAAAagatct-3′)^7^ into the *δ51-LucII* vector. *FLAG-mTead4, Gal4-mTead4c* and *HA-mYap* plasmids are described in Nishioka N *et al*. and Ota M *et al*.^3^,^32^. *FLAG-mNotch1ΔE (deletion of extracellular domain)-Venus* and *Myc-mSsrp1* constructs were kindly provided by Dr. Saga^78^ and Dr. Murata^79^, respectively. *hSBNO1, Myc-mNICD1 (V1744-K2531*), *FLAG-mRbpj* and *mSpt16-VSVG* expression plasmids were constructed by PCR amplification using appropriate sets of primers.

### Transfection of DNA and siRNA, and luciferase assay

Twenty-four hours before transfection, 5 × 10^4^ 293 T or E14Tg2a ES cells/well were seeded in 24-well plates. DNA mixtures of *luciferase* reporter (0.05 μg/well), effector (0.1~0.5 μg/well), CMV-βgal (0.05 μg/well, as an internal control), and pcDNA3.0 (to keep total amounts of transfected DNAs constant) were mixed with three volumes of polyethylenimine or XtremeGENE-HP (Roche) for 293 T cell, or 1.5 ul/well PLUS reagent with 3.75 ul/well Lipofectamine LTX (Invitrogen) for ES cell, and then added to cells. For siRNA experiments, cells were transfected with mixtures of 2 μl/well XtremeGENE-siRNA (Roche) and 1.5 μl/well of 20 μM *Sbno1* stealth siRNA (Invitrogen, mixture of #HSS124121, #HSS124122 and #HSS182932) or negative control Low GC duplex (Invitrogen) (the final concentration of siRNA was 50 nM). The medium was changed 24 hours after the siRNA treatment, and then DNA was transfected with three volumes of XtremeGENE-HP (Roche). Transfected cells were cultured for 48 hours and then lysed to measure luciferase activities using a LMAX II luminometer (Molecular Devices). β-Galactosidase activity was measured using an iMark microplate reader (BioRad) to normalize the luciferase activities. All transfections were performed in triplicate, and independently repeated at least three times, which gave reproducible results. For the luciferase assay on mechanically stretched cells, 5 × 10^4^ or 1 × 10^5^ 293 T cells were seeded on fibronectin-coated 2 × 2 cm stretch silicone chambers 24 hours before transfection. DNA mixtures of the luciferase reporter (0.05 μg/well), effector (0.1 μg/well), CMV-βgal (0.05 μg/well, as an internal control) and empty pcDNA3.0 (to keep total amounts of transfected DNAs constant) were mixed with three volumes of polyethylenimine and then added to 293 T cells. Transfected cells were cultured for 24 hours. The medium was changed 2 hours before cell stretching. Transfected cells were stretched (1 Hz, 5% stretch, 2 hours) (STB Cell Stretching System, Strex, Osaka, Japan) and then rested in non-stretched conditions for 2 hours. Stretched cells were lysed to measure luciferase activity as described above.

### Generation of *Sbno1* knockout mouse

The *Sbno1* knockout mouse line was generated by Ozgene Pty. Ltd. as follows. A loxP-fused region of exon 7 (709 bp) was amplified by PCR from C57BL/6 genomic DNA and subcloned upstream of an FRT-flanked *Pgk* promoter-*Neo* resistance gene-polyA (*PGK-neo*)-loxP cassette. The 5′- (4,261 bp) and 3′-homology arms (2,851 bp) were also amplified by PCR from C57BL/6 genomic DNA. These arms were subcloned upstream of the loxP-exon7 fragment and downstream of the PGK-neo cassette, respectively (Supplementary Fig. 2a). This targeting vector was electroporated into Bruce 4 ES cells (derived from C57BL/6). Targeted ES clones were selected by G418 treatment, and analyzed by Southern blot analysis with 5′ and 3′ probes (Supplementary Fig. 2a,b). Correct clones were injected into blastocysts to produce chimeric mice. After breeding the F1 generation, the loxP-flanked exon 7 and *PGK-Neo* cassette were deleted by crossing with the *Oz-Cre* mouse strain (Ozgene), which possesses ubiquitous Cre activity. *Sbno1* wild type (Wt), floxed (f) and knockout (Δ) alleles were genotyped by PCR with F; 5′-AGACTGGTGGTGTGCAGTACC-3′ and R1, 5′-GAAAGAAGGCTCGGTGGCTAA-3′ or R2, 5′-CACCACTGCATCAGGGTGAC-3′ primers. F and R1 primers amplify 840 and 250 bp fragments from Wt and knockout alleles, respectively. F and R2 primers amplify 350 and 420 bp fragments from Wt and floxed alleles, respectively (Supplementary Fig. 2a,c). All animal experiments were performed in accordance with institutional guidelines, and full details of the animal experimental protocols were approved and ethical permission was granted by Animal Care Committee of Tohoku University.

### RNA injection and embryo culture

Zygotes or two-cell stage embryos were collected from oviducts by flushing with M2 medium (Sigma). *EGFP-hSBNO1-Wt* or -*E437Q* RNA was synthesized by mMESSAGE mMACHINE kit (Thermo) and purified RNA (100 ng/ul) was injected to the zygotes. Embryos were cultured in a drop of KSOM (ARK resource) covered by mineral oil at 37 °C, 5% CO_2_. Pictures were taken using an MZ16 microscope (Leica) and a DFC310FX digital camera (Leica), and fluorescent images were taken by FV1000 (Olympus).

### RT-PCR

Total RNAs of preimplantation embryos were extracted with Trizol reagent (Invitrogen) and cDNAs synthesized using SuperScript III reverse transcriptase (Invitrogen), according to the manufacturer’s protocol. PCR was performed with Blend Taq (Toyobo) for 40 cycles of 94 °C for 30 sec, 60 °C for 30 sec, and 72 °C for 30 sec. Primers used are shown in Supplementary Table.

### Immunohistochemistry, cell death detection and *in situ* PLA

Preimplantation or cultured embryos were collected and fixed in 4% paraformaldehyde for 30 minutes. After washing with 0.2% lamb serum in PBS (PBSS), embryos were permeabilized by 0.2% Triton-100 in PBS for 30 minutes, and then blocked with 2% lamb serum in PBS. The following primary antibodies were diluted to optimal concentrations in blocking buffer and incubated with embryos overnight at 4 °C: α-Sbno1 (Abcam, #ab122789), α-β-catenin (BD Transduction, #610153), α-phospho-Histone H3 (Cell Signaling, #9701), α-Oct3/4 (MBL, #PM048), α-Nanog (ReproCell, #RCAB002P-F), α-Cdx2 (Biogenex, #MU392A-UC), α-Tbr2 (Eomesodermin) (Abcam, #ab23345), α-Keratin 8 (Developmental Studies Hybridoma Bank, #TROMA-I), α-Yap (Cell Signaling, #4912), α-Tead4 (Abcam, #ab58310), and α-Rbpj (Cell Signaling, #5313). After washing the embryos wish PBSS, secondary antibodies (Alexa Fluor 488, 546 or 594 goat anti-mouse or anti-rabbit IgG, Molecular probes) were diluted 1/1000 in PBSS before incubation for 1 hour. For detection of cell death, embryos were incubated in the TUNEL reaction mixture of the *in situ* cell death detection kit AP (Roche) for 1 hour at 37 °C. Duolink *In situ* PLA was performed according to the manufacture’s protocol (Sigma). Nuclei were stained with 4′,6-diamidino-2-phenylindole in PBS or Topro-3 in 40% glycerol/PBS. Fluorescent images were captured using an FV1000 (Olympus) or TCS-SP5 (Leica) confocal microscope at 3–4-μm optical sections.

### Co-immunoprecipitation assay

293 T cells were seeded in a 10-cm dish at a density of 2 × 10^6^ cells/dish 24 hours before transfection. Cells were transfected with 3–5 μg of expression plasmids using XtremeGENE-HP (Roche) according to the manufacturer’s protocol. Cells were harvested 48 hours after transfection and lysed in 500 μl lysis buffer (10 mM HEPES pH 7.6, 250 mM NaCl, 0.1% NP40, 5 mM EDTA and protease inhibitors). After homogenization and brief sonication, the lysates were centrifuged, and the supernatant was subjected to immunoprecipitation with the following antibodies (2 μg); α-Myc (9E10; Santa Cruz, #sc-40), α-HA (F-7; Santa Cruz, #sc-7392), α-FLAG (DDDDK; MBL, #M185), α-V5 (MBL, #M167-3) or α-GFP (MBL, #598). Twenty microliters of protein-G PLUS-Agarose beads (Santa Cruz, #sc-2002) were added to the lysates, and incubated for 30 minutes at 4 °C. The beads were washed four times with 1 ml lysis buffer, and dissolved in 40 μl 2xSDS (sodium dodecyl sulfate) sample buffer. Immunoprecipitates were separated by SDS polyacrylamide gel electrophoresis, and transferred to polyvinylidene fluoride membrane. Target proteins were probed with the following primary antibodies: α-Myc (Cell Signaling, #2278), α-HA (Cell Signaling, #3274), α-FLAG (DDDDK; MBL, #PM020), α-V5 (MBL, #PM003), α-GFP (MBL, #598) or α-VSVG (MBL, #563), and then goat anti-mouse or anti-rabbit horseradish peroxidase–conjugated secondary antibody. The membrane was reacted with ECL Prime Western Blotting Detection Reagent (GE Healthcare), and chemiluminescent signals were visualized with ImageQuant LAS 4000 mini (GE Healthcare).

## Additional Information

**How to cite this article**: Watanabe, Y. *et al*. Notch and Hippo signaling converge on Strawberry Notch 1 (Sbno1) to synergistically activate *Cdx2* during specification of the trophectoderm. *Sci. Rep.* **7**, 46135; doi: 10.1038/srep46135 (2017).

**Publisher's note:** Springer Nature remains neutral with regard to jurisdictional claims in published maps and institutional affiliations.

## Supplementary Material

Supplementary Information

Supplementary Movie
