# Supplementary material for: Notch and Hippo signaling converge on Strawberry Notch 1 (Sbno1) to synergistically activate Cdx2 during specification of the trophectoderm
Source: Sci Rep. 2017 Apr 12;7:46135. doi: 10.1038/srep46135 (PMC5389439; doi:10.1038/srep46135)
Supplement: Supplementary Information [file srep46135-s1.pdf]

Supplementary Information

Notch and Hippo signaling converge on Strawberry Notch 1 (Sbno1) to synergistically activate *Cdx2* during specification of the trophectoderm

Yusuke Watanabe[1,2], Miyasaka Y. Kota[1], Atsushi Kubo[1], Yasuyuki S. Kida[3], Osamu Nakagawa[4], Yoshikazu Hirate[5,7], Hiroshi Sasaki[5,6], Toshihiko Ogura[1,*]

[1] Department of Developmental Neurobiology, Institute of Development, Aging and Cancer, Tohoku University, 4-1 Seiryo, Aoba, Sendai, Miyagi 980-8575, Japan
[2] Department of Molecular Physiology, National Cerebral and Cardiovascular Center Research Institute, 5-7-1 Fujishiro-dai, Suita, Osaka, 565-8565, Japan
[3] Biotechnology Research Institute for Drug Discovery, National Institute of Advanced Industrial Science and Technology (AIST), 1-1-1 Umezono, Tsukuba, Ibaraki 305-8568, Japan
[4] Department of Molecular Physiology, National Cerebral and Cardiovascular Center Research Institute, Suita, Osaka, Japan
[5] Department of Cell Fate Control, Institute of Molecular Embryology and Genetics, Kumamoto University, 2-2-1 Honjo, Chuo-ku, Kumamoto 860-0811, Japan
[6] Laboratory for Embryogenesis, Graduate School of Frontier Biosciences, Osaka University, 1-3 Yamadaoka, Suita, Osaka 585-0871, Japan
[7] Present address: Center for Experimental Animals, Tokyo Medical and Dental University, 1-5-45 Yushima, Bunkyo-ku, Tokyo 113-8510, Japan

* Contact
Corresponding author email: toshihiko.ogura.c3@tohoku.ac.jp

Supplementary Methods

Generation of tamoxifen-inducible *Sbno1* knockout embryonic stem (ES) cells

E2.5 *Sbno1*$^{flox/flox}$ embryos were collected, and cultured in KSOM supplemented with 1μM PD0325901 and 3μM CHIR99021 (2i) for 2 days, and then transferred the embryos into 2i medium (DMEMF12/Neurobasal medium supplemented with 1x N2, 1x B27, 1x sodium pyruvate, 1x nonessential amino acids, 1x L-glutamine, LIF, 0.1mM β-mercaptoethanol and 2i) {Ying, 2008 #102} on mouse embryonic fibroblasts as feeder cells. After 6 days culture, ES cell colonies were observed. The ES cells were passaged several times to addapt feeder-free condition. To introduce *MerCreMer* in *Sbno1*$^{flox/flox}$ ES cells, *pPiggyBac-CAG-MerCreMer-IH* and *PBase (PiggyBac Transposase)* plasmid constructs (kindly provided by Dr. Niwa H, Kumamoto Univ.) were transfected to the ES cells by Lipofectamine 2000 (Invitrogen), and then the *MerCreMer*-integrated *Sbno1*$^{flox/flox}$ cells were selected by Hygromycin (75μg/ml). Recombination of *Sbno1*^{flox} to *Sbno1*$^{\Delta}$ allele by addition of tamoxifen (1μg/ml) was confirmed by PCR and western blot analysis (supplementary figure 3a,b).

Acknowledgements

We thank Dr. H Niwa (Kumamoto Univ.) for protocol and advices for ES cell and PiggyBac plasmids.

Supplementary Figure 1

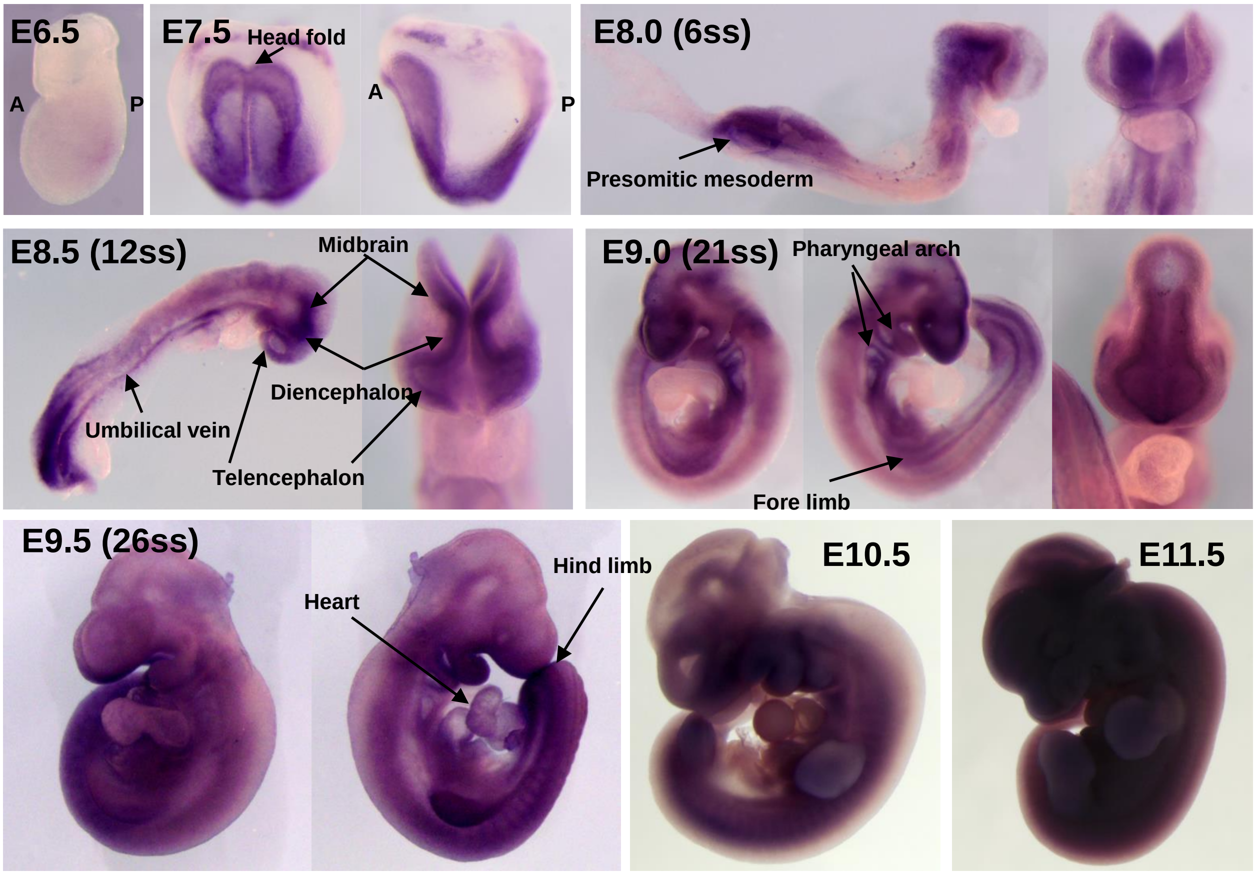

Supplementary Figure 1. Whole-mount in situ hybridization of *Sbno1* transcripts in post-implantation embryos. *Sbno1* expression initiates in the head and presomitic mesoderm at E7.5–E8.0, and then the expression becomes evident in umbilical vein, pharyngeal arch and limbs at E8.5–E9.0. After E9.5, the expression is expanded to the whole body, and becomes ubiquitous by E11.5.

Supplementary Figure 2

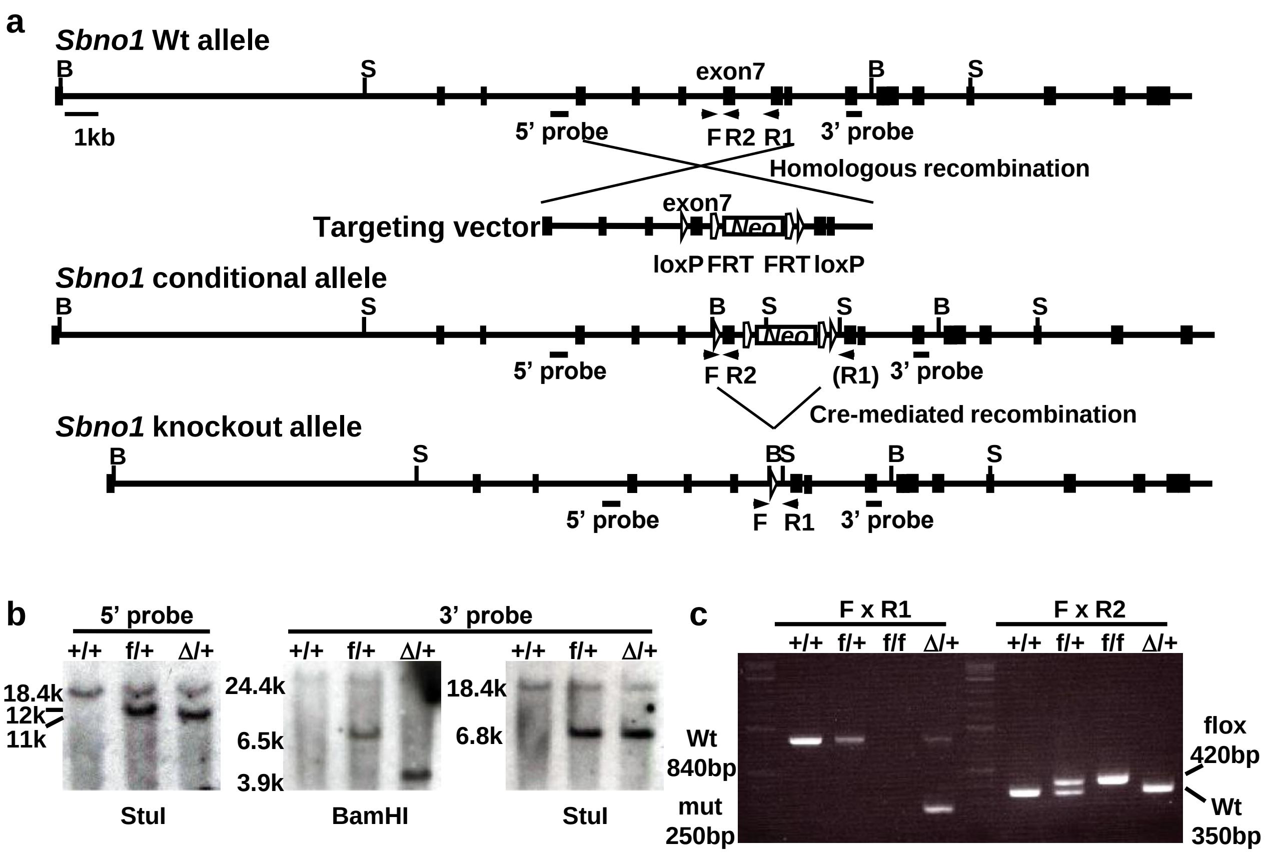

Supplementary Figure 2. Generation of *Sbno1* knockout embryos. (a) Strategy to generate a *Sbno1* knockout mouse, in which exon 7 of *Sbno1* was targeted. This results in a premature termination of Sbno1. (b,c) The targeted conditional (flox; f) and deleted (Δ) alleles were verified by Southern blot analysis with two restriction enzymes (*Stu*I and *Bam*HI), 5′- and 3′- probes (b) and also by PCR amplification with an appropriate set of primers (c).

Supplementary Figure 3

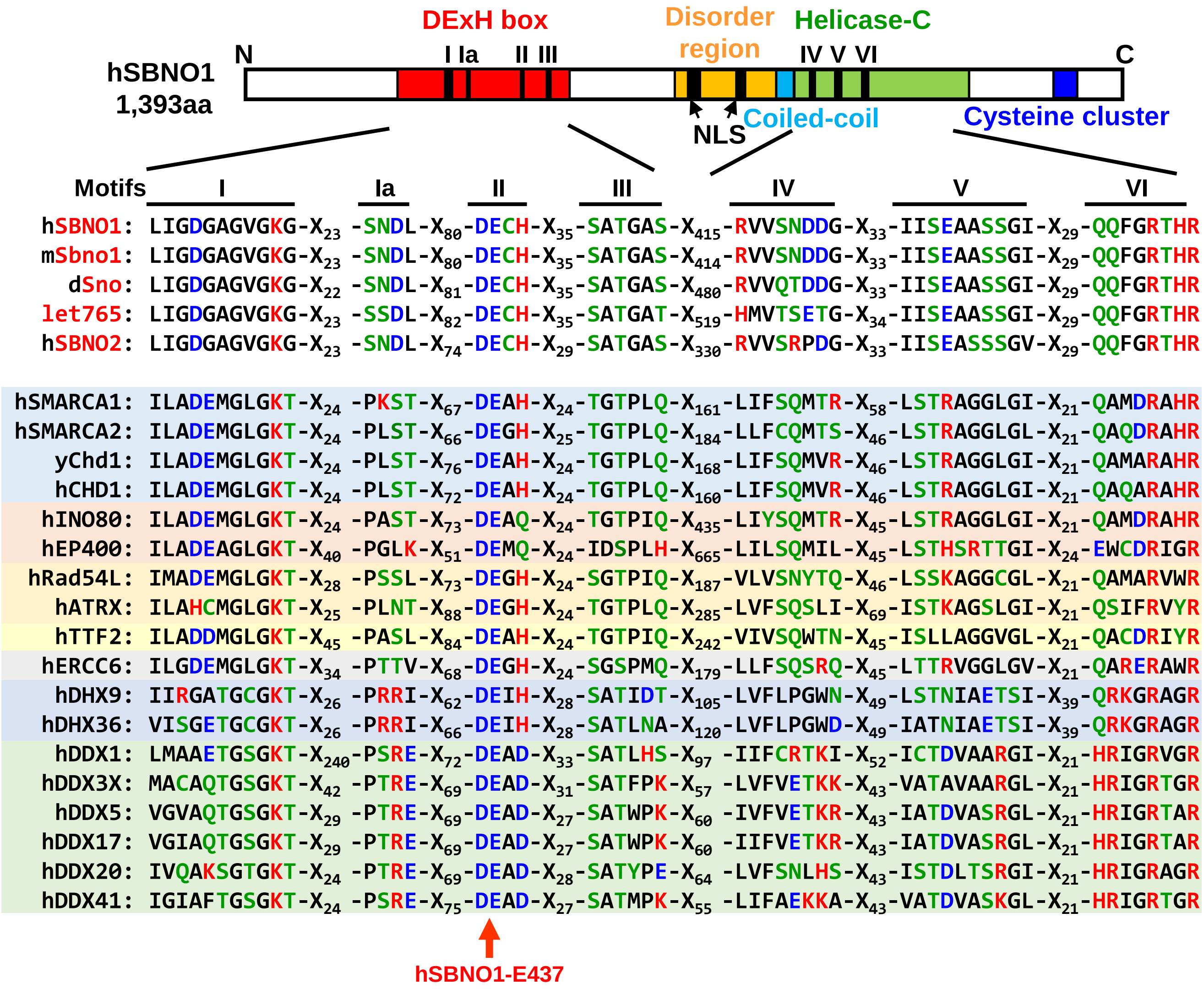

Supplementary Figure 3. Comparison of protein structures among Sbno/Sno and SF2 family members. Amino acid sequences of the seven conserved motifs (I to VI) and spacing between them are shown. The sequences of Sbno/Sno (human SBNO1, mouse Sbno1, *Drosophila* Sno, *C. elegans* let765, and human SBNO2) are aligned with other members of the SF2 family, including Snf2-like (hSMARCA1, hSMARCA2, yChd1, hCHD1), Swr1-like (hINO80, hEP400), Rad54-like (hRad54L, hATRX), Rad5/16-like (hTTF2), SSo1653-like (hERCC6), DExH box helicases (hDHX9, hDHX36), and DExD box helicases (hDDX1, hDDX3X, hDDX5, hDDX17, hDDX20, hDDX41). Residues colored in black, blue, red and green are hydrophobic, acidic, basic and others, respectively. Position of hSBNO1 E437, a critical amino acid for the transcriptional activation shown in Figures 3-5, is indicated (hSBNO1-E437).

Supplementary Figure 4

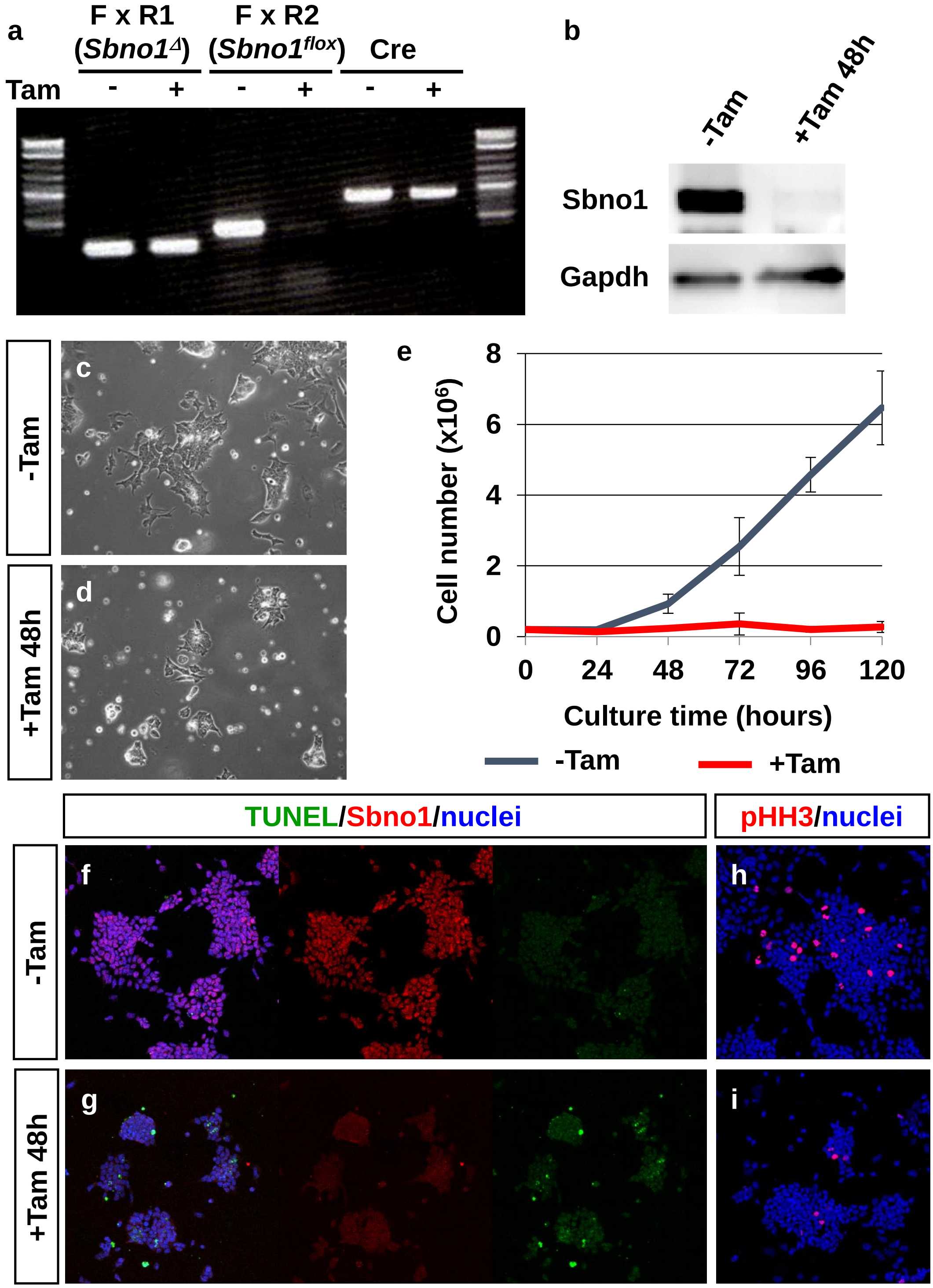

Supplementary Figure 4. Generation of tamoxifen-inducible *Sbno1* knockout embryonic stem cell and its phenotype. (a,b) Tamoxifen (Tam)-inducible *Sbno1* KO ES cell line (*Sbno1$^{flox/flox}$;MerCreMer*) is established (a), and deletion of *Sbno1^{flox}* allele and diminishment of Sbno1 protein are confirmed by PCR (a) and Western blot (b), respectively, after 48h of Tam treatment. (c-i) *Sbno1* KO ES cells cannot increase cell number (c-e), and cell death is increased and proliferation is decreased, shown by TUNEL assay (f,g) and pHH3 staining (h,i), respectively.

Supplementary Figure 5

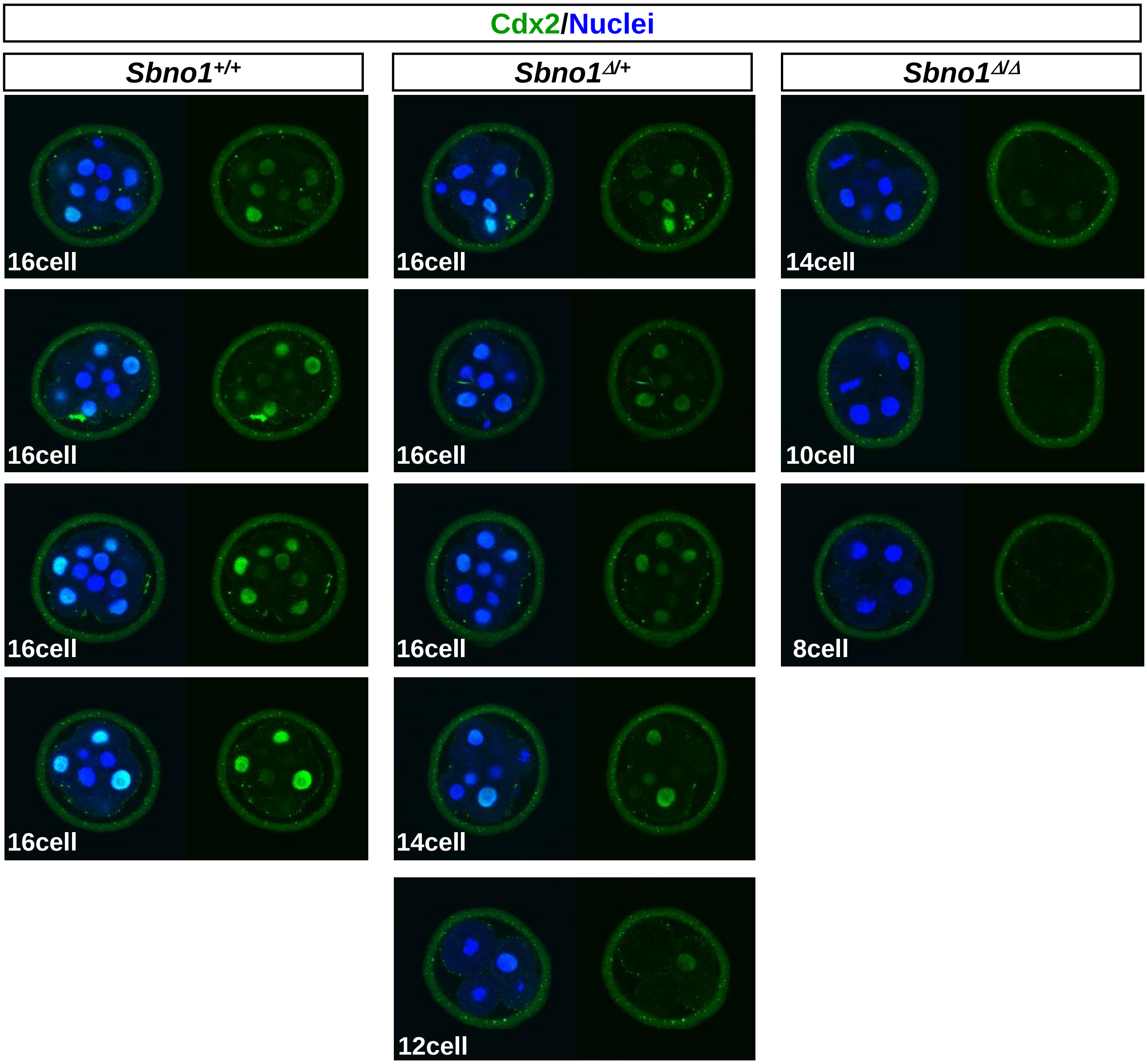

Supplementary Figure 5. Cdx2 expression in control (*Sbno1*$^{+/+}$ and *Sbno1*$^{\Delta/+}$) and *Sbno1*$^{\Delta/\Delta}$ embryos at E2.5. *Sbno1*$^{\Delta/\Delta}$ embryos delay the development compare to control *Sbno1*$^{+/+}$ and *Sbno1*$^{\Delta/+}$ embryos, and no or little expression of Cdx2 is observed at E2.5.

Supplementary Figure 6

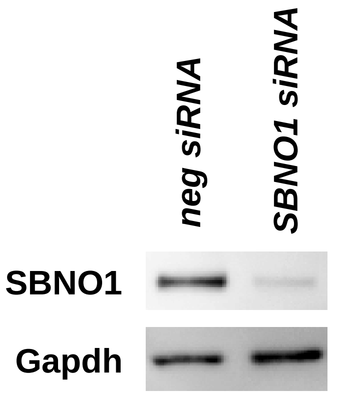

Supplementary Figure 6. Knockdown efficiency of *SBNO1* siRNA. Western blot analysis was performed with an anti-SBNO1 antibody on extracts prepared from 293T cells over-expressing *SBNO1 siRNA* and negative control siRNA (*neg siRNA*).

Supplementary Figure 7

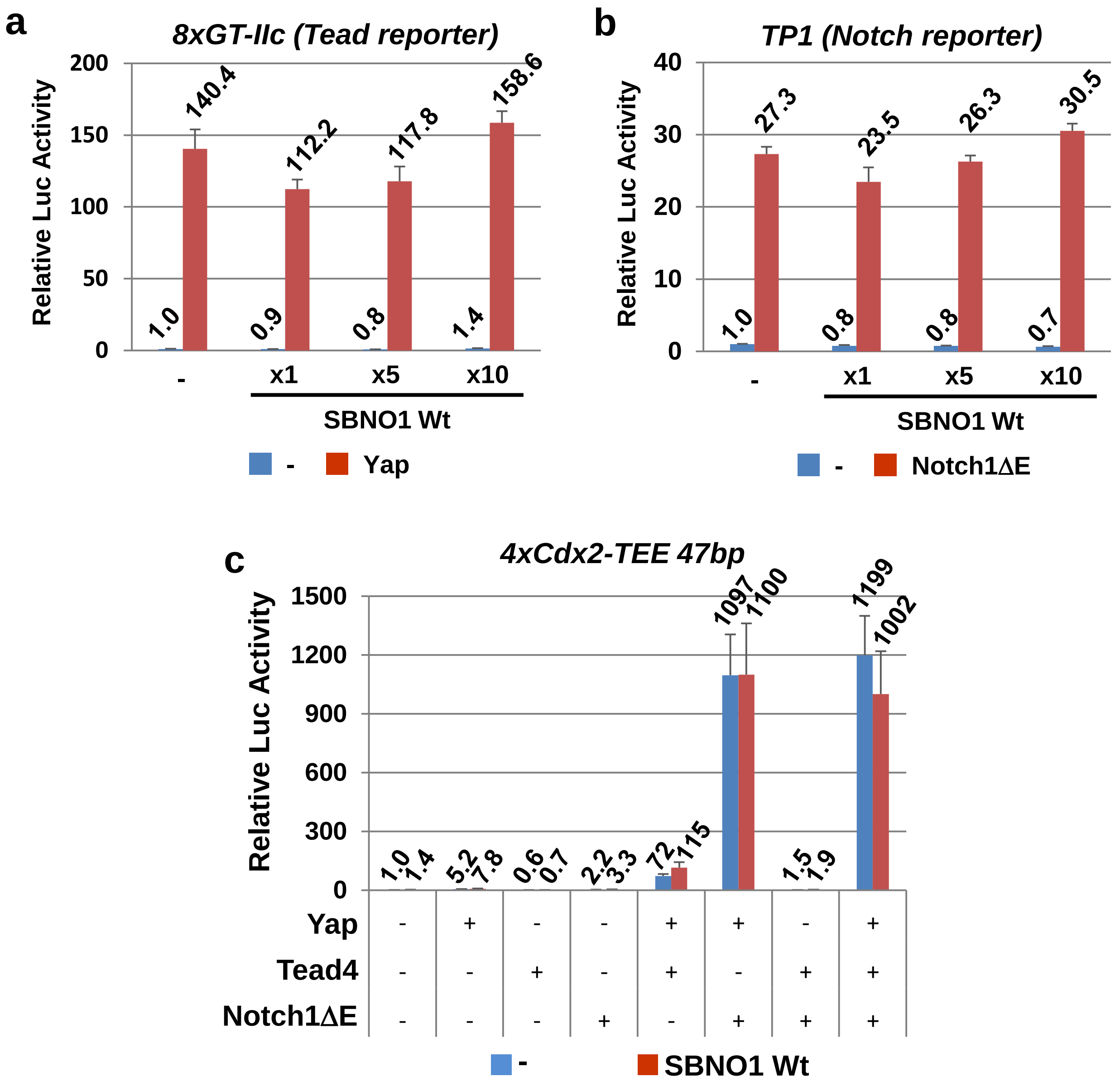

Supplementary Figure 7. Effect of SBNO1 Wt on transcriptional activation of Tead and Notch reporters and the *Cdx2* trophectoderm enhancer (*Cdx2*-TEE) in 293T cells. Addition of SBNO1 Wt expression plasmid did not influence transcriptional activities of 8xGT-IIc and TP1 reporters (a,b) and 4x*Cdx2*-TEE (c) in 293T cells.

Supplementary Figure 8

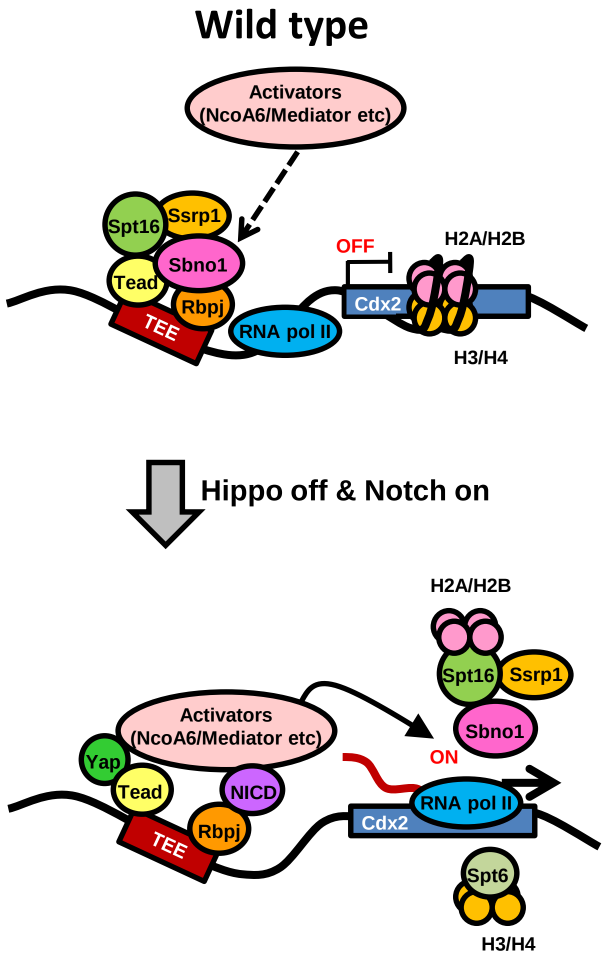

Supplementary Figure 8. Hypothetical model of Sbno1 actions on *Cdx2* transcriptional activation. In the wild type embryos, when Hippo signaling is active and Notch signaling is inactive, Sbno1 interacts with Tead4, Rbpj and the FACT complex (Ssrp1 and Spt16). When Hippo signaling is inactive and Notch signaling is active, Yap and Notch-intracellular domain (NICD) translocate into the nucleus to bind to their DNA-binding partners (Tead4 and Rbpj, respectively) and recruit transcriptional co-activators on the TEE of *Cdx2*. Sbno1 is released from this complex to facilitate uncoiling of nucleosomal DNA of *Cdx2* and consequent transcriptional elongation by RNA polymerase II (RNA polII). In the absence of Sbno1, the FACT complex loses an access to Tead4 and Rbpj. Hence, the transcriptional elongation halts by the histone complex, which is not removed by Sbno1 and the FACT complex. We do not exclude a possibility that Sbno1 might help association of activators to the Yap/Tead4 and NICD/Rbpj complexes. In the absence of Sbno1, recruitment of co-activators might be inefficient.

Supplementary Figure 9

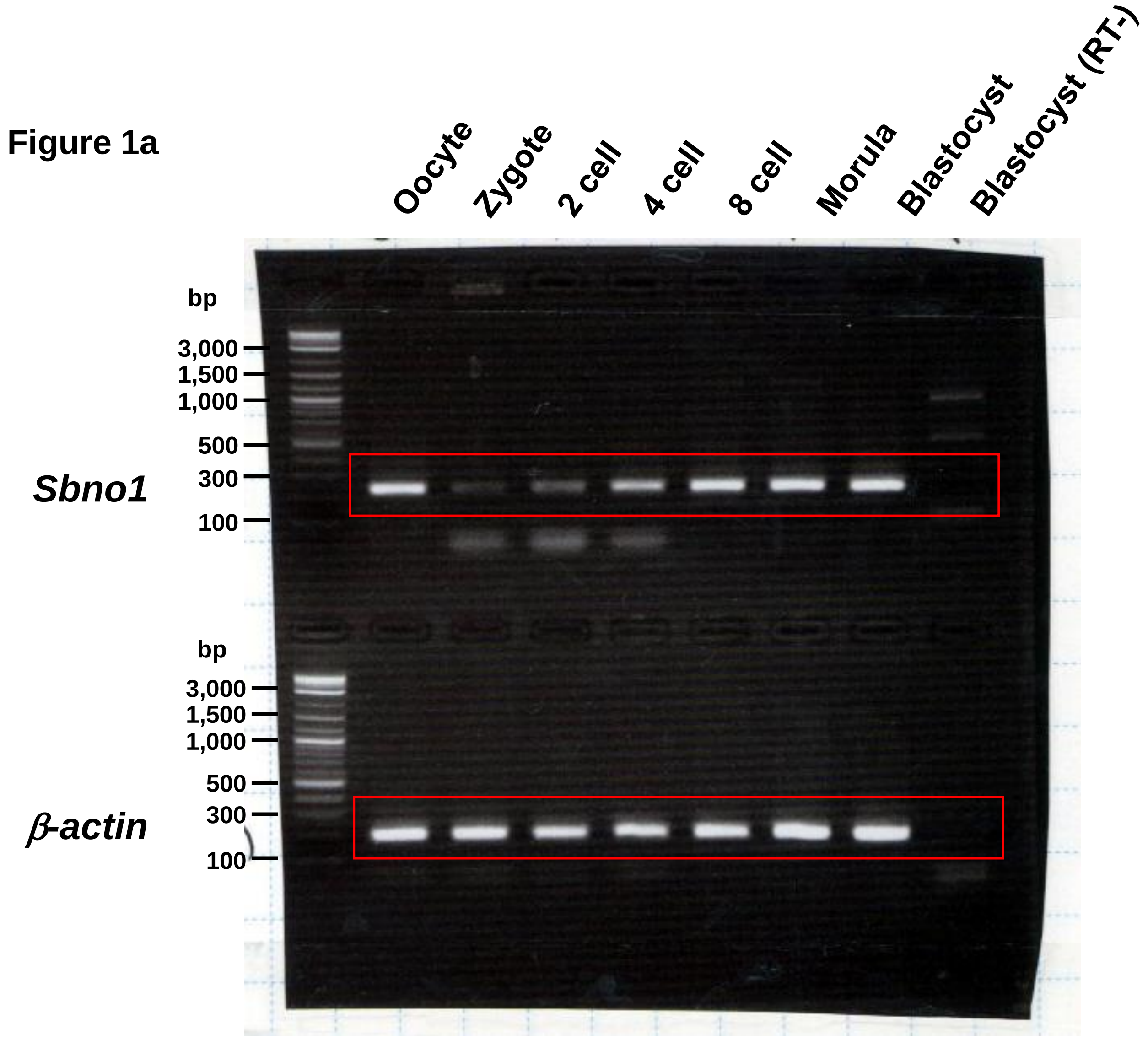

Supplementary Figure 9. Uncropped scans of Figure 1a. Full length DNA gel electrophoresis of Figure 1a to detect *Sbno1* and *β-actin* expression.

Supplementary Figure 10

Figure 2m

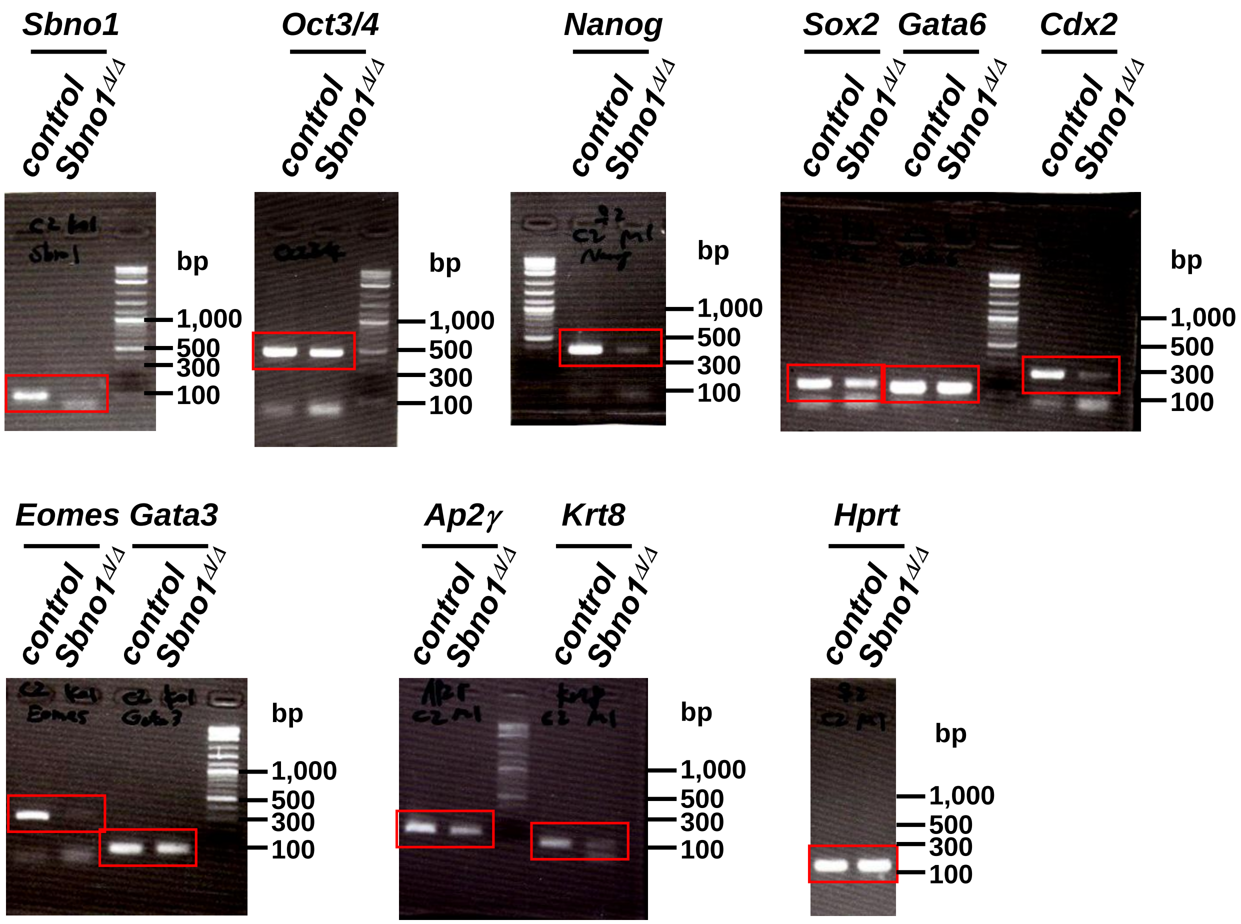

Figure 2w

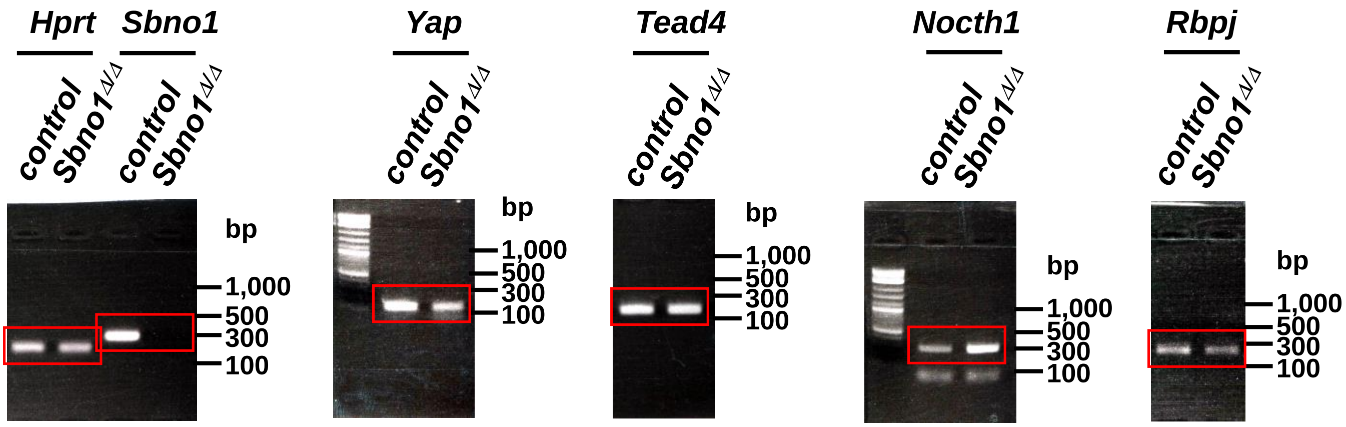

Supplementary Figure 10. Uncropped scans of Figure 2m and 2w. Full length DNA gel electrophoresis of Figure 2m and 2w to detect *Sbno1*, *Oct3/4*, *Nanog*, *Sox2*, *Gata6*, *Cdx2*, *Eomes*, *Gata3*, *Ap2γ*, *Krt8*, *Yap*, *Tead4*, *Notch1*, *Rbpj* and *Hprt* expression.

Supplementary Figure 11

Figure 6a

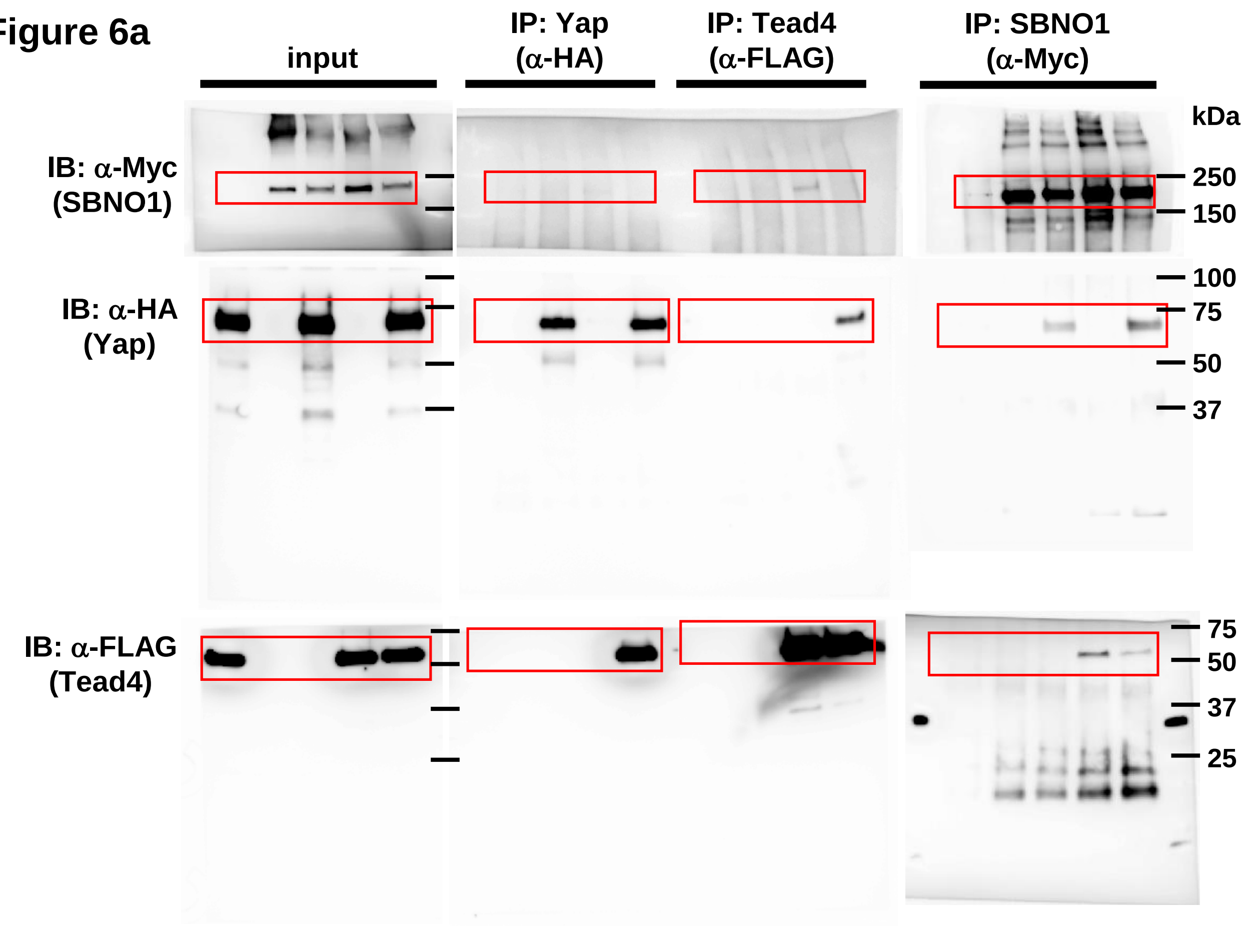

Figure 6b

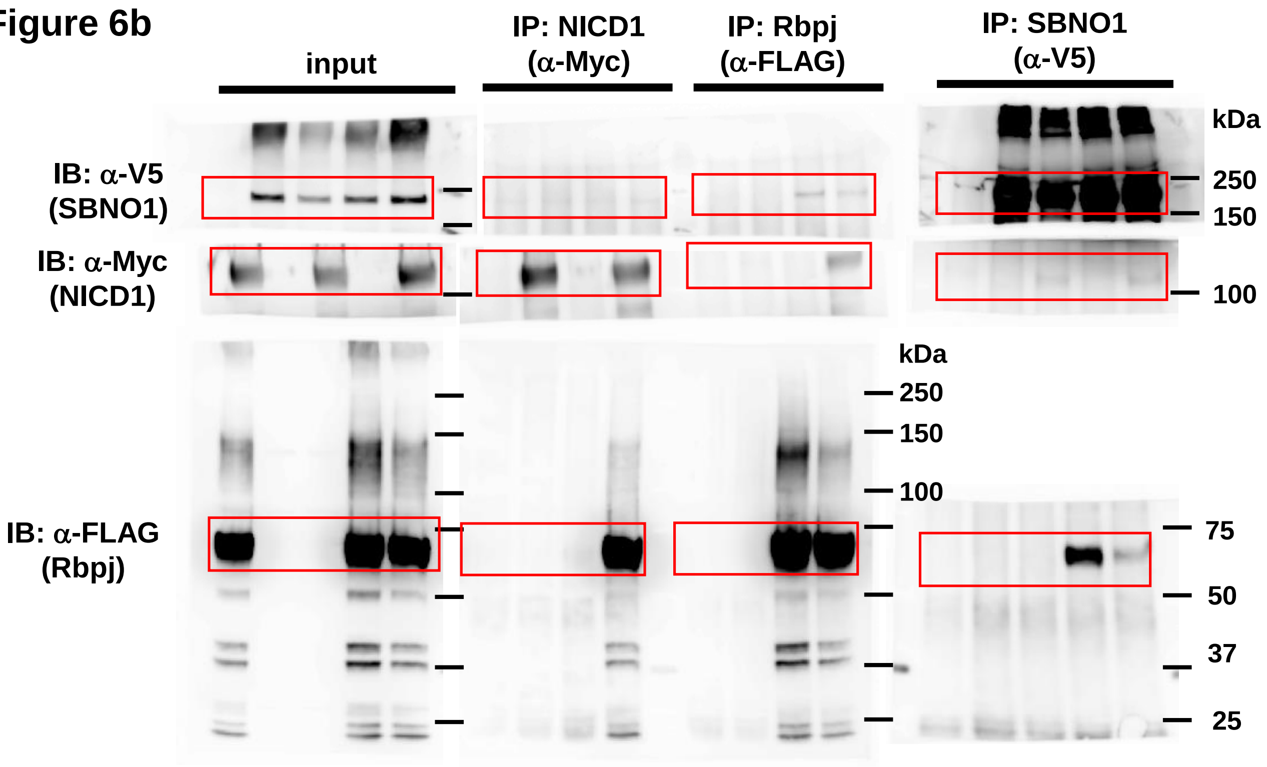

Supplementary Figure 11. Uncropped scans of Figure 6a and 6b. Uncropped scans of western blots of Figure 6a and 6b to Myc-SBNO1, HA-Yap, FLAG-Tead4, V5-SBNO1, Myc-NICD1 and FLAG-Rbpj expression.

Supplementary Figure 12

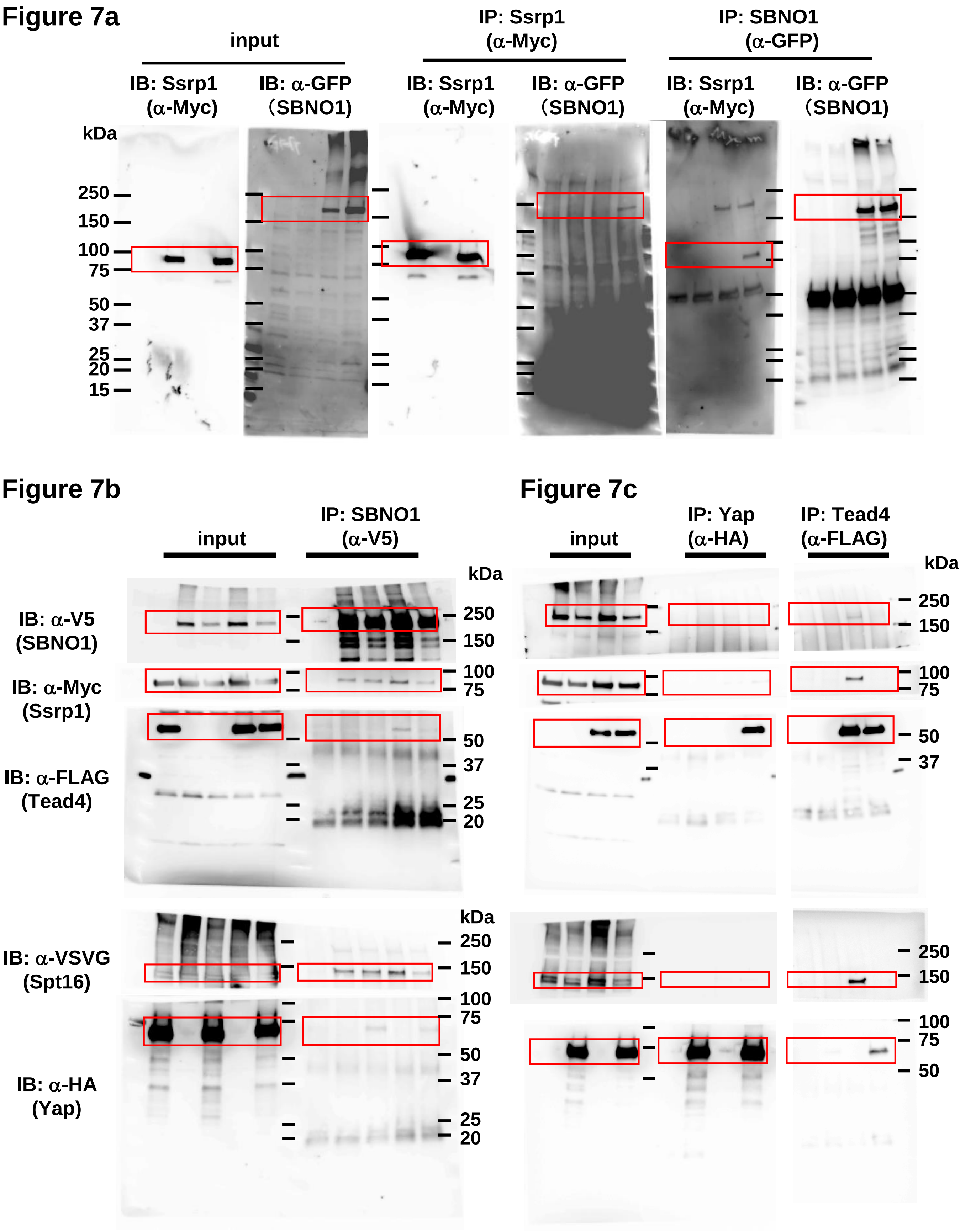

Supplementary Figure 12. Uncropped scans of Figure 7a, 7b and 7c. Uncropped scans of western blots of Figure 7a, 7b and 7c to GFP-SBNO1 and Myc-Ssrp1 (7a) or V5-SBNO1, Myc-Ssrp1, FLAG-Tead4, VSVG-Spt16 and HA-Yap expression (7b, 7c).

Supplementary Table

RT-PCR primers

Gene	Forward (5' to 3')	Reverse (5' to 3')	Reference
Sbno1	AAAATTGGCCTGCGTCACC	CTTTACCCACACCAGCACCA	
Oct3/4	CTGAGGGCCAGGCAGGAGCACGAG	CTGTAGGGAGGGCTTCGGGCACTT	[1]
Sox2	AACTTTTGTCCGAGACCGAGA	CGGGAAGCGTGTACTTATCCTT	[1]
Nanog	GTTTGCCTAGTTCTGAGGAAGCA	ATGGAGGAGAGTTCTTGCATCTG	[1]
Gata6	CGGTCTCTACAGCAAGATGAATG	CTACGCCATAAGGTAGTGGTTGTG	
Cdx2	TCCCTAGGAAGCCAAGTGAAAAC	TGCTGCTGCTTCTTCTTGATTTT	[1]
Eomes	CCTGGTGGTGTTTTGTTGTG	TTTAATAGCACCGGGCACTC	[2]
Gata3	GCCTGCGGACTCTACCATAAA	ATGTCCCTGCTCTCCTTGCT	
Krt8	CATCAAGAAGGATGTGGACGAA	TGCAACTCACGGATCTCCTCT	
Ap2γ	GGCTGACTATTTAACGAGACCACA	CAGTTTTGTATGTTCGGCTCCA	
Yap	TCAGACAACAACATGGCAGGA	CCCATCCATCAGGAAGAGGT	
Tead4	GTTGGAGTTCTCGGCTTTCCT	CTTGTCATAGATTTGGCGGATG	
Notch1	CCCGCTGTGAGATTGATGTT	CGTTCTTGCATGGTGTGCT	
Rbpj	TTCTATGGCAACAGCGATGAC	TTGTGAACTGGCGTGGAAA	
Hprt	CAGCCCCAAAATGGTTAAGGT	GTCAAGGGCATATCCAACAACA	
β-actin	CTTCTTTGCAGCTCCTTCGTT	ACCCATTCCCACCATCACA	

RT-PCR primers used in Figures 1A and 2M and W are shown.

Supplementary References

1. Ema M, *et al.* Kruppel-like factor 5 is essential for blastocyst development and the normal self-renewal of mouse ESCs. *Cell Stem Cell* **3**, 555-567 (2008).

2. Niwa H, *et al.* Interaction between Oct3/4 and Cdx2 determines trophectoderm differentiation. *Cell* **123**, 917-929 (2005).

Supplementary Movie

Supplementary Movie 1. In vitro culture of two-cell stage littermates from the *Sbno1Δ/+* intercross. Embryos were cultured in a drop of KSOM covered by mineral oil at 37ºC, 5% CO_2. Sbno1 mutants did not develop to the blastocyst stage and collapsed.
